# Bacterioplankton Diversity and Distribution in Relation to Phytoplankton Community Structure in the Ross Sea Surface Waters

**DOI:** 10.3389/fmicb.2022.722900

**Published:** 2022-01-27

**Authors:** Angelina Cordone, Giuseppe D’Errico, Maria Magliulo, Francesco Bolinesi, Matteo Selci, Marco Basili, Rocco de Marco, Maria Saggiomo, Paola Rivaro, Donato Giovannelli, Olga Mangoni

**Affiliations:** ^1^Department of Biology, University of Naples Federico II, Naples, Italy; ^2^Department of Life Sciences, DISVA, Polytechnic University of Marche, Ancona, Italy; ^3^National Research Council, Institute of Marine Biological Resources and Biotechnologies CNR-IRBIM, Ancona, Italy; ^4^Stazione Zoologica Anton Dohrn, Naples, Italy; ^5^Department of Chemistry and Industrial Chemistry, University of Genoa, Genoa, Italy; ^6^Department of Marine and Coastal Science, Rutgers University, New Brunswick, NJ, United States; ^7^Marine Chemistry and Geochemistry Department, Woods Hole Oceanographic Institution, Woods Hole, MA, United States; ^8^Earth-Life Science Institute, Tokyo Institute of Technology, Tokyo, Japan; ^9^Consorzio Nazionale Interuniversitario delle Scienze del Mare (CoNISMa), Rome, Italy

**Keywords:** bacterial diversity, bacterioplankton, phytoplankton, Ross Sea, Antarctica

## Abstract

Primary productivity in the Ross Sea region is characterized by intense phytoplankton blooms whose temporal and spatial distribution are driven by changes in environmental conditions as well as interactions with the bacterioplankton community. However, the number of studies reporting the simultaneous diversity of the phytoplankton and bacterioplankton in Antarctic waters are limited. Here, we report data on the bacterial diversity in relation to phytoplankton community structure in the surface waters of the Ross Sea during the Austral summer 2017. Our results show partially overlapping bacterioplankton communities between the stations located in the Terra Nova Bay (TNB) coastal waters and the Ross Sea Open Waters (RSOWs), with a dominance of members belonging to the bacterial phyla Bacteroidetes and Proteobacteria. In the TNB coastal area, microbial communities were characterized by a higher abundance of sequences related to heterotrophic bacterial genera such as *Polaribacter* spp., together with higher phytoplankton biomass and higher relative abundance of diatoms. On the contrary, the phytoplankton biomass in the RSOW were lower, with relatively higher contribution of haptophytes and a higher abundance of sequences related to oligotrophic and mixothrophic bacterial groups like the Oligotrophic Marine Gammaproteobacteria (OMG) group and SAR11. We show that the rate of diversity change between the two locations is influenced by both abiotic (salinity and the nitrogen to phosphorus ratio) and biotic (phytoplankton community structure) factors. Our data provide new insight into the coexistence of the bacterioplankton and phytoplankton in Antarctic waters, suggesting that specific rather than random interaction contribute to the organic matter cycling in the Southern Ocean.

## Introduction

Antarctica and the Southern Ocean are central to Earth’s climate and oceanic circulation systems (Convey and Peck, 2019). Primary productivity in this region is characterized by intense phytoplankton blooms whose temporal and spatial distribution are driven by different environmental conditions, although the mechanisms regulating these processes are still poorly known (Moore and Abbott, 2000; Deppeler and Davidson, 2017). In this context, the Ross Sea is one of the most productive sectors in Antarctica (Smith and Gordon, 1997; Arrigo et al., 1999), with an annual productivity averaging ∼180 g C m^−2^ year^−1^ (Arrigo et al., 2008). Phytoplankton blooms here have been well-studied, with the dominance of diatoms and haptophytes presenting different temporal and spatial patterns (Smith et al., 2014; Mangoni et al., 2017). In the last years, however, changes in phytoplankton blooms and dynamics that contrast the classical Antarctic paradigm have been observed as for example the dominance of *Phaeocystis antarctica* in stratified coastal waters during summer, the high levels of biomass in an area usually considered an High Nutrient Low Chlorophyll (HNLC), or the presence of relative higher percentage of minor functional groups contrasting the classical Antarctic paradigm (DiTullio et al., 2000; Phan-Tan et al., 2018; Mangoni et al., 2019; Bolinesi et al., 2020b). All together, these observations require a revaluation of the phytoplankton-bacteria interaction, which could be playing a key role in structuring the trophodynamics in this area (Bertrand et al., 2007, 2015).

The assumption that the Antarctic Ocean microbial communities were generally species poor has been re-discussed in recent years, since many results suggested that microbial diversity is significantly higher than previously recognized (Murray and Grzymski, 2007; Wilkins et al., 2013; Silvi et al., 2016). In the last decade, researchers have demonstrated that in Antarctic pelagic food webs micro-eukaryote dynamics significantly contribute to the structuring of the prokaryotic community (Kirchman et al., 2001; Wilkins et al., 2013; Delmont et al., 2014 and references therein) and in turn prokaryotic diversity can influence phytoplankton productivity under certain conditions (Bertrand et al., 2007, 2015). Prokaryotic communities contribute to or dominate several key ecosystem processes, including primary production, the turnover of biogenic elements, the mineralization of the organic matter, and the degradation of xenobiotics and pollutants (Cole, 1982; Azam et al., 1983; Croft et al., 2005; Azam and Malfatti, 2007; Falkowski et al., 2008; Sher et al., 2011).

Early work in Antarctic waters revealed that bacterial biomass might represent up to 30% of total microbial biomass in coastal areas (Fiala and Delille, 1992). In Antarctica, free-living marine microbial community composition can differ significantly between locations at relatively small spatial and temporal scales, responding to environmental variations of temperature, salinity, nutrients, or the presence of oceanic fronts (see for example, the Supplementary Material of Lozupone and Knight, 2007; Raes et al., 2018). For example, in Terra Nova Bay (TNB), substantial differences in terms of bacterial assemblages have been observed between coastal and offshore stations and along the water column (Celussi et al., 2009). Heterotrophic members of the Alphaproteobacteria and Gammaproteobacteria class of the Proteobacteria have been reported as dominant phylotypes in Antarctic waters (Giovannoni et al., 2005; Murray and Grzymski, 2007; Wilkins et al., 2013), and studies in these and other marine ecosystems indicate that bacterial growth is frequently dependent on phytoplankton-derived DOM (Church et al., 2000; Morán et al., 2001, 2002; Piquet et al., 2011; Ducklow et al., 2012; Kim et al., 2014). A broad diversity among the class Flavobacteria has been also reported in different sub-areas of the Southern Ocean (Abell and Bowman, 2005). According to Piquet et al. (2011), melt water stratification and the transition to non-stabilized Antarctic surface waters may have an impact not only on micro-eukaryotes but also on bacterial community composition, with a shift from an Alpha- and Gammaproteobacteria to a Cytophaga–Flavobacterium–Bacteroides-dominated community under mixed conditions. Some studies found an unusual presence of strictly anaerobic Epsilonproteobacteria (now reclassified as phylum Campylobacterota) in the bottom of sea ice (Gentile et al., 2006), probably as a consequence of the oxygen decay and sulfide accumulation, caused by high degradation rates of sympagic diatoms by aerobic and anaerobic heterotrophs (Brierley and Thomas, 2002).

Shifts in the Eukaryotic plankton communities have been often reported as rapidly followed by a shift in the bacterial community (Billen and Becquevort, 1991; Piquet et al., 2011), with a series of phytoplankton–bacterial interactions resulting in both positive and negative feedback loops (Bertrand et al., 2015). For example, in temperate waters phytoplankton blooms in spring and summer induce changes in bacterioplankton community structure (Fuhrman et al., 2006; Teeling et al., 2016; Chafee et al., 2018). These community dynamics have been shown to be recurrent, indicating a phytodetritus-driven seasonality and suggesting that the phytoplankton-prokaryotic interactions in surface waters are more sophisticated than previously thought (Seymour et al., 2017). Mechanisms addressing the nature of the mutualistic interaction between phytoplankton and bacterioplankton communities have been proposed over time. For example, the exchange of phytoplankton exudates and bacteria-produced cobalt containing vitamin B_12_ represents one of the best studied feedback loops (Bertrand et al., 2007, 2011; Bolinesi et al., 2020b). The production and release of vitamin B_12_ by bacteria, in fact, depends on the degradation of phytoplankton exudates, establishing a complex feedback mechanism between prokaryotic and phytoplanktonic communities by heterotrophic bacteria (Fang et al., 2017). Similar trophic interaction between phytoplankton and bacterioplankton in the marine ecosystem, and especially in polar regions, might be more common than previously known. The bacterial-phytoplankton interaction may thus evolve in a series of complex relationship, affecting directly or indirectly the micronutrient availability and co-limitation (e.g., iron, cobalt, and vitamin B_12_; Bertrand et al., 2007; Tagliabue et al., 2017; Bolinesi et al., 2020a) as well as nutrient uptake (Amin et al., 2015; Bertrand et al., 2015). Yet, despite this the number of studies reporting the simultaneous diversity of the phytoplankton and bacterioplankton community in the Antarctic waters is comparatively few (Di Poi et al., 2013; Flaviani, 2017; Richert et al., 2019). In this study, we analyzed the bacterial diversity in relation to phytoplankton in the surface waters of TNB and Ross Sea, providing new insight into the coexistence of the two communities in Antarctic waters.

## Materials and Methods

### Sampling Procedure and Study Site

**Figure 1:**
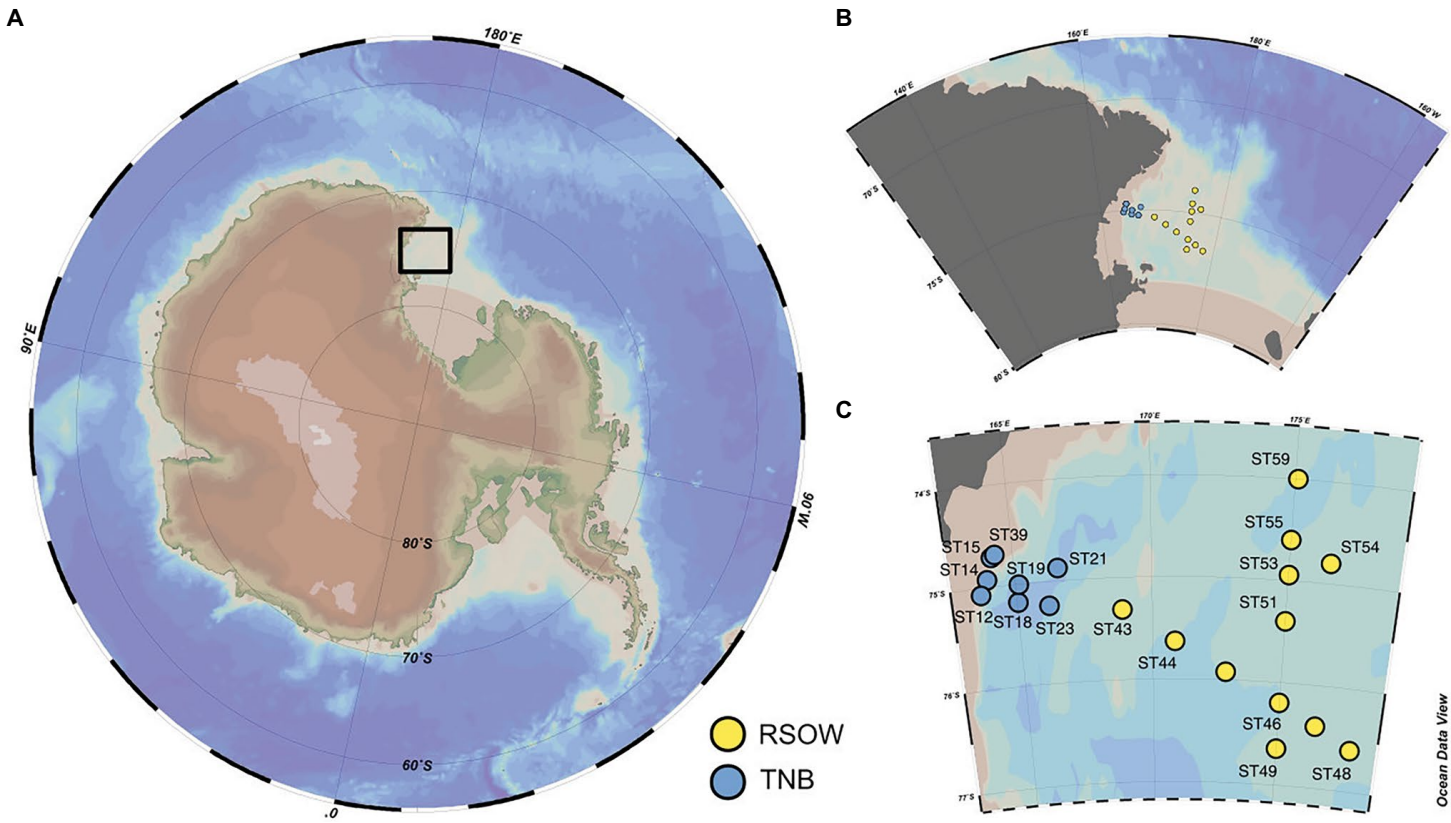
Study location and distribution of the sampled stations across the Ross Sea. **(A)** Location of the study site within the Ross Sea. **(B,C)** Details of the spatial distribution of the sampled stations and their division in Terra Nova Bay (TNB) stations proximal to the sea-ice border and the coast and the Ross Sea Open Waters (RSOW) stations, located further off-shore. This division and color scheme is consistent throughout the paper.

Seawater samples were collected either at the deep chlorophyll maximum or at ~20 m depth in stations where the fluorescence profile showed a rather homogeneous distribution of chlorophyll-a (Chl-a) in the upper layer (Table 1). Sampling activities were carried out on the R/V Italica during the Austral Summer 2017 (between 13 and 30 Jan 2017), in the framework of Plankton biodiversity and functioning of the Ross Sea ecosystems in a changing Southern Ocean (P-ROSE) and CDW Effects on glacial mElting and on Bulk of Fe in the Western Ross sea (CELEBeR) projects – Italian National Antarctic Program – funded by the Ministry of Education, University and Research (MIUR). The area of investigation falls within two different zones (Figure 1) of the Ross Sea, the coastal area of TNB and the Ross Sea Open Water (RSOW). Water samples were collected using a carousel sampler (Sea-Bird Electronics 32) equipped with 24 12-L Niskin bottles and a conductivity–temperature-depth (CTD) instrument (9/11 Plus; Sea-Bird Electronics), along a transect from the coastal area of TNB to the oper Ross Sea (Figure 1C), crossed by a north-south aligned secondary transect carried out in the RSOW area. For the bacterial diversity analysis, at each station, 500 ml of seawater was collected from the Niskin bottle and filtered shipboard onto a 0.22 μm filter (Whatman, 47 mm diameter) successively stored at −80°C and transported back to the lab. For the analysis of total phytoplankton biomass, 500 ml of seawater was filtered shipboard through 0.45 μm GF/F filter (Whatman, 47 mm diameter). For the determination of size classes of phytoplankton, 500 ml of seawater was prefiltered on board onto a 20 or 2 μm net, and the flow through filtered on a 0.45 μm GF/F (Whatman 47 mm diameter) obtaining two size fractionated subsamples. All GF/F filters were preserved frozen at −80°C and transported back to the lab for analysis (see below). The contribution of the different size classes was calculated as the difference between total phytoplankton biomass the two size fractions to provide micro- (>20 μm), nano- (between 20 and 2 μm), and pico- (<2 μm) phytoplankton biomass. For the determination of phytoplankton functional groups by chemotaxonomic criteria, 2 L of seawater were filtered on board onto 0.45 μm GF/F filter (Whatman, 47 mm diameter) and stored at −80°C. For the phytoplankton counts and taxonomic analysis, water samples were immediately fixed with 4% CaCO_3_ buffered formalin solution. For the analyses of macronutrient concentrations (NO_3_^−^, NO_2_^−^, NH_4_^+^, Si(OH)_4_, and PO_4_^3−^), water samples were taken directly from the Niskin bottles and stored at −20°C in 20 ml low-density polyethylene containers until laboratory analysis. Monthly mean sea surface Chl-a concentrations in log(mg m^−3^) at 4 km resolution derived from the MODIS-AQUA sensor (Satellite remote sensing Ocean color data) were downloaded from the European Data Portal (Melin, 2013).

**Table 1:** Summary of the sampled stations and main environmental variables.

Station	Sampling area	Latitude (°N)	Longitude (°E)	Bottom depth (m)	Sampling depth (m)	Salinity (PSU)	Temperature (°C)	Chlorophyll-a (μg/L)
ST12	TNB	−75.07186	163.70473	867	35	34.46	−0.9	2.5
ST14	TNB	−74.9274	163.9883333	342	20	34.28	1.7	3.8
ST15	TNB	−74.71124	164.23106	498	20	34.36	1.7	2.4
ST18	TNB	−75.17703	165.04118	1,054	15	34.1	1	2.4
ST19	TNB	−75.0045667	165.1273167	925	20	34.67	0.3	2.4
ST21	TNB	−74.8748767	166.5811933	886	30	34.1	−0.1	1.3
ST23	TNB	−75.23705	166.1794517	852	15	34	0.5	2
ST39	TNB	−74.7128833	164.2245833	497	25	34.48	−0.4	1.3
ST43	RSOW	−75.3133617	168.8873467	351	28	34.18	−0.2	1.7
ST44	RSOW	−75.6297333	170.8507833	568	39	34.42	−0.6	1.1
ST45	RSOW	−75.9229083	172.8276267	571	28	34.37	−0.1	0.7
ST46	RSOW	−76.1994917	174.9960983	568	28	34.42	−0.4	1.6
ST47	RSOW	−76.40035	176.4959233	409	45	34.45	−0.4	1.2
ST48	RSOW	−76.6003167	177.9980833	292	42	34.47	−0.6	1.4
ST49	RSOW	−76.6460167	174.9964333	425	26	34.33	−0.2	2.4
ST51	RSOW	−75.4	175.0048333	297	33	34.32	0	0.3
ST53	RSOW	−74.9452	175.0106	326	35	34.17	−0.2	1.1
ST54	RSOW	−74.8008333	176.5028	299	28	34.28	−0.1	1.7
ST55	RSOW	−74.6025833	175.0045167	439	15	34.15	−0.3	2.2
ST59	RSOW	−73.99957	175.10031	579	34	34.17	−0.4	2

### Phytoplankton Community Structure and Biomass

Frozen samples were processed in Italy for the determination of Chl-a and phaeopigments (Phaeo-a) content (used as proxy for phytoplankton biomass) using a solution of 90% acetone according to Holm-Hansen et al. (1965), with a spectrofluorometer (Shimadzu) checked daily with a Chl-a standard solution (from Sigma-Aldrich). HPLC pigments separation was performed on an Agilent 1100 HPLC according to the method outlined in Vidussi et al. (1996) as modified by Mangoni et al. (2017). The following biomarker pigments were used as chemotaxonomic descriptors: alloxanthin (cryptophytes), chlorophyll b (chlorophytes), prasinoxanthin (prasinophytes), 19′-butanoyloxyfucoxanthin (pelagophytes), fucoxanthin (diatoms), 19′-hexanoyloxyfucoxanthin (haptophytes), peridinin (dinophytes), and zeaxanthin (Cyanobacteria). The contribution of the main phytoplankton groups to the total Chl-a was estimated on the basis of the concentrations of biomarker pigments using the chemical taxonomy software CHEMTAX (Mackey et al., 1996). The phytoplankton species composition and cells abundance were determined following the Uthermöhl method (Utermöhl, 1931), according to which at least 400 cells were counted per sample with an inverted light microscope (LM) Zeiss Axiovert Observerz. One at 400× magnification and used to estimate specific group abundance. In order to better visualize the shifts in the phytoplankton community, we computed a diatoms to haptophytes ratio (D/H ratio), calculated using the relative abundance of diatoms and haptophytes as: (diatoms/haptophytes)/(diatoms + haptophytes).

Phytoplankton photosynthetic efficiency was estimated using the maximum photochemical quantum yields of PS II (*F*v/*F*m), representing the initial maximum efficiency of photons captured by open PSII reaction centers. The maximum photochemical quantum yields of PS II were measured using a Phyto_PAM II compact unit (waltz) as described in Bolinesi et al. (2020b). All samples were acclimated in the dark before analysis to minimize the non-photochemical dissipation of excitation, and measurements were blank corrected by filtering the sample through a 0.2 μm filters. For determining *F*v/*F*m, samples were illuminated with a saturating pulse, and the ratio was calculated using the formula *F*v/*F*m = (*F*m − *F*_0_)/*F*m as previously described.

### Nutrient Analysis

Inorganic nutrients were analyzed using a five-channel continuous flow autoanalyzer (Technicon Autoanalyser II), according to the method described by Hansen et al. (1983) adapted to the available instrumentation. Briefly, samples flow was controlled by a multi-channel peristaltic pump, which regulates the flow rate of samples and reagents throughout the analytical procedures. The sample flow was segmented with air bubbles to enhance mixing of reagents and sample, and to reduce smearing. The sample-reagent mixture reacts chemically to produce color in proportion to the concentration of the nutrient in the sample, and is analyzed in a flowcell using a phototube as detector.

### Community DNA Extraction

Total community DNA was extracted as previously reported (Giovannelli et al., 2013) with slight modifications. Briefly, each filter was washed with 1.7 ml of extraction buffer solution [100 mM NaCl (pH 8.0), 20 mM EDTA, and 50 mM Tris-HCl (pH 8.0)] added with 10 μl Proteinase K (1 mg/ml) and incubated at 37°C for 1 h with occasionally mixing. A 100 μl volume of 10% sodium dodecyl sulfate (SDS) was added to each sample, followed by incubation at 55°C for 1 h. The liquid phase was collected and centrifuged for 15 min at 10,000×*g* to separate cellular debris from nucleic acids. The supernatant was extracted twice with an equal volume of phenol, followed by a precipitation with 2.5 volume of ethanol 100% and 0.1 volumes of sodium acetate (3 M) for 12 h at −20°C. The DNA was collected by centrifugation, washed in 70% cold ethanol, air dried, and resuspended in 50 μl of sterile distilled water. The integrity of DNA was assessed spectrophotometrically and by PCR amplification of the 16S rRNA gene using the primes Ribo-For (5′-AGTTTGATCCTGGCTCAG-3′) and Ribo-Rev (5′-CCTACGTATTACCGCGGC-3′) (Fakhry et al., 2008). The PCR products were visualized on 1% agarose gel stained with ethidium bromide.

### 16S rRNA Gene Sequencing

Partial 16S rRNA gene sequences were obtained using primer pair Probio_Uni/Probio_Rev, which target the V3 region of the 16S rRNA gene sequence (Milani et al., 2013). 16S rRNA gene sequencing was performed using a MiSeq platform (Illumina) at the DNA sequencing facility of GenProbio srl^1^ according to the protocol previously reported (Milani et al., 2013). The used primers were tested using Silva TestPrime 1.0 against the SSU r132 allowing 0–2 mismatched and showed a good coverage and specificity for the Bacteria domain (96.1% coverage and 73.3% specificity), while having a low specificity for the Archaea (91.3% coverage and 15.2% specificity). Given the low specificity for Archaea of the primer set used in this study, we have obtained results for the bacterial population only.

### Bioinformatics and Statistical Analyses

All sequences were imported in R, and analyzed with the DADA2 package (Callahan et al., 2016). Following the package guidelines, quality plots were performed to check the sequences’ quality. Post-QC reads were trimmed using the filterAndTrim command [truncLen = c(155,145), maxN = 0, maxEE = c(2,2), truncQ = 2, rm.phix = TRUE, trimLeft = 17, and trimRight = 15]. After this step, a parametric error model, based on the convergence between the estimation of error rate and the inference of the sample composition, was performed. Paired-end reads were merged and exact Amplicon Sequence Variants (ASVs) inferred using the dada algorithm. Chimeric sequences were removed and prokaryotic taxonomy assigned using the naive Bayesian classifier method against the Silva Database (r132; https://www.arb-silva.de/documentation/release-132/). ASVs abundance table obtained from DADA2 was further processed in R using *Phyloseq*, *Vegan*, and *Microbiome* packages (McMurdie and Holmes, 2013; Lahti and Shetty, 2017; Oksanen et al., 2020). Sequences are available through the European Nucleotide Archive (ENA) with bioproject accession number ERP129169. A complete R script containing all the steps to reproduce our analysis is available at https://github.com/giovannellilab/Cordone_et_al_Ross_Sea with DOI: https://doi.org/10.5281/zenodo.4784454.

After the Phyloseq object creation, low abundance ASVs (less than three reads across the dataset), Mitochondria, Chloroplast, Eukaryotes sequences, and potential contaminants (Sheik et al., 2018) were removed. The resulting dataset was represented by 703 unique ASVs and 535,009 reads. ASVs counts were normalized to the median library size across the dataset. Diversity analyses were carried out using the Phyloseq package (McMurdie and Holmes, 2013) with relative abundance set to 100% after the removal of sequences described above. Top abundance ASVs and Genera were defined has having a cumulative relative abundance above 0.1% in our dataset. The alpha diversity was investigated using both the Simpson and Shannon diversity index among the two sampled areas. The beta diversity was investigated using the UNIFRAC and Jaccard diversity index as implemented in the vegan package (Oksanen et al., 2012). Both the abundance weighted and unweighted version of the index was used. The resulting similarity matrix was plotted using non-metric multidimensional scaling techniques implemented in the ordination command of Phyloseq. The resulting ordination was used to investigate correlations with environmental and phytoplankton variables using the *envfit* and *ordisurf* functions in vegan. Collinearity among the predictors was checked using a Pearson correlation matrix. The linearity of the correlation between the rate of change in the beta diversity and the variables identified as significant by the *envfit* function was checked by plotting the non-metric multidimensional scaling (nMDS) axis against the variable. Statistically significant differences in the distribution of abundant bacterial genera were tested using the Chi-square test. Co-correlation networks were calculated as a pairwise distribution of each ASV across the entire dataset using Spearman rank correlation and different ρ cutoff selected for network plotting using the igraph package (Csardi and Nepusz, 2006).

## Results

### Phytoplankton Community and Physical–Chemical Properties of the Water Column

Water temperature ranged between −0.3 and 1.7°C (mean value of 0.7 ± 0.8°C) in TNB, and between −0.6 and 0.1°C (mean value of −0.3 ± 0.2°C) in the RSOW (Kruskal–Wallis, *p* < 0.05). Salinity ranged between 34.0 and 34.4 (mean of 34.2 ± 0.2) in TNB, while showed less variability in RSOW with values ranging between 34.2 and 34.4 (mean of 34.3 ± 0.1; Kruskal–Wallis, nonsignificant). Further details on physical water column properties are described in the T/S diagrams previously published in Bolinesi et al. (2020b) highlighting differences between subsystems. In TNB, dissolved inorganic nitrogen (DIN) concentrations, as sum of nitrate, nitrite, and ammonium, ranged between 11.09 and 26.37 μmol/L, PO_4_^3−^ ranged between 0.79 and 1.82 μmol/L, while Si(OH)_4_ ranged between 35.77 and 49.95 μmol/L. In RSOW, DIN ranged between 18.61 and 25.52 μmol/L, PO_4_^3−^ between 1.24 and 1.63 μmol/L, and Si(OH)_4_ between 42.41 and 55.68 μmol/L (Table 2).

**Table 2:** Concentrations of nutrients in the sampled stations at the sampling depth.

Station	Sampling area	DIN (μmol/L)	PO_4_^3−^ (μmol/L)	Si(OH)_4_ (μmol/L)	N/P
ST12	TNB	26.37	1.82	39.78	14.49
ST14	TNB	11.09	0.83	35.77	13.37
ST15	TNB	11.81	0.81	37.62	14.6
ST18	TNB	12.32	0.79	37.8	15.68
ST19	TNB	17.47	1.23	49.48	14.22
ST21	TNB	21.07	1.46	49.95	14.44
ST23	TNB	15.16	0.89	39.99	17.05
ST39	TNB	22.43	1.63	45.52	11.64
ST43	RSOW	22.01	1.29	42.41	16.45
ST44	RSOW	23.35	1.57	48.88	14.01
ST45	RSOW	22.18	1.63	51.41	13.21
ST46	RSOW	18.61	1.62	55.68	10.65
ST47	RSOW	16.79	1.47	50.3	10.37
ST48	RSOW	18.26	1.49	49.97	11.1
ST49	RSOW	19.67	1.42	53.77	13.25
ST51	RSOW	20.58	1.59	54.87	12.2
ST53	RSOW	24.23	1.24	47.08	18.65
ST54	RSOW	23.83	1.48	45.41	14.47
ST55	RSOW	24.45	1.47	44.78	16.04
ST59	RSOW	25.72	1.63	55.36	14.85

**Figure 2:**
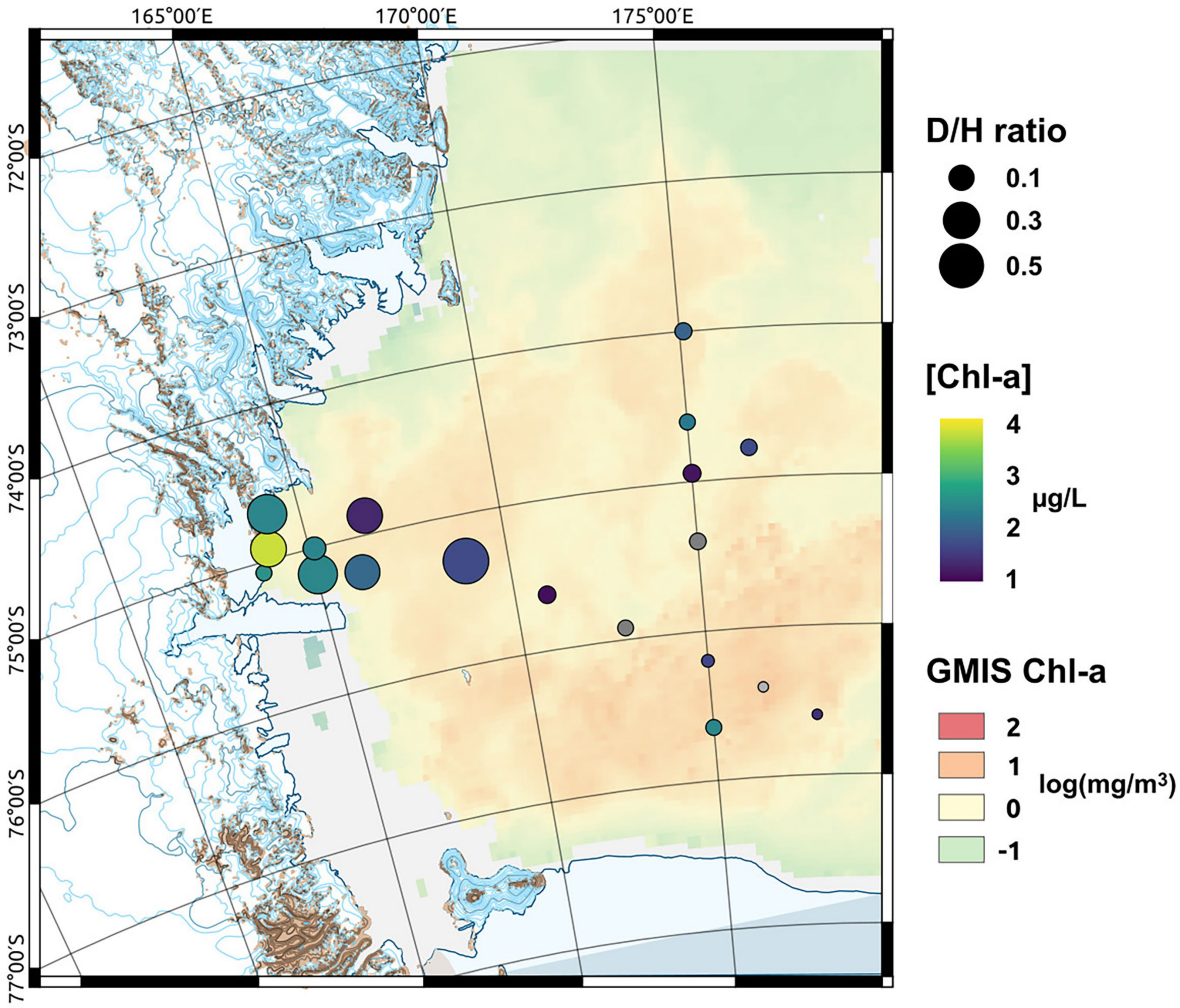
Map of the sampled area showing the monthly mean sea surface Chlorophyll-a (Chl-a) concentration [in log(mg m^−3^) at 4 km resolution GMIS Chl-a] derived from satellite observation, the measured Chl-a concentrations measured at the sampled depth ([Chl-a]) and the diatoms to haptophytes ratio (D/H ratio) describing the shift in the phytoplankton community composition.

The distribution of phytoplankton showed strong differences in terms of total biomass (measured as Chl-a concentrations, Table 1) and main functional groups between the two areas (Table 3). In TNB Chl-a ranged between 1.26 and 3.75 μg/L, with diatoms strongly dominating the community with a mean abundance of 87%. In the RSOW values of Chl-a ranged between 0.29 and 2.39 μg/L, with diatoms accounting for 62% and haptophytes for 32% of the total biomass (Table 4; Figure 2). As concerns *F*v/*F*m in TNB values ranged between 0.2 (stations ST18 and ST23) and 0.46 (coastal station ST12), with a mean of 0.31 ± 0.1. In RSOW, values ranged between 0.23 (station ST43) and 0.45 (station ST48), with a mean of 0.45 ± 0.07. The analyses of phytoplankton abundance in terms of cells per liter revealed that in TNB the most abundant diatoms species were *Pseudo-nitzschia* spp. (3.1 × 10^5^–1.6 × 10^6^ cells/L), *Fragilariopsis* spp. (5.8 × 10^5^–5.5 × 10^6^ cells/L), and *Chaetocheros* spp. (1.3 × 10^4^–2.8 × 10^5^ cells/L). Other diatoms were on average 7.4(±5) × 10^5^ cells/L. *Phaeocystis antarctica* ranged between 1.6 × 10^5^ and 2.9 × 10^6^ cells/L, while the total number of dinoflagellates ranged between 1.2 × 10^5^ and 2.4 × 10^5^. Other phytoplankton groups (*Cryptophyceae*, *Crysophyceae*, *Chlorophyceae*, *Prasinophyceae*, and small flagellates) reached the maximum of 3.2 × 10^6^ cells/L. Choanoflagellates have been reported here within phytoplankton, with a total number of cells of 1.1 × 10^4^ cells/L. In the RSOW, *P. antarctica* was the most abundant species (3.2 × 10^5^–5.5 × 10^6^ cells/L). Among diatoms, the most abundant species in RSOW were *Pseudo-nitzschia* spp. (1.7 × 10^5^–2 × 10^6^ cells/L), *Fragilariopsis* spp. (1.6 × 10^5^–2.3 × 10^6^ cells/L), and *Chaetocheros* spp. (7.9 × 10^4^–3.1 × 10^5^ cells/L). Other diatoms were on average 2.6(±2) × 10^5^ cells/L, while the total number of dinoflagellates showed a mean value of 1.1(±1) × 10^5^. Other groups (*Cryptophyceae*, *Crysophyceae*, *Chlorophyceae*, *Prasinophyceae*, and small flagellates) showed a mean value of 9.8(±3) × 10^5^ cells/L. It must be noted that during the cruise, a high concentration of Choanoflagellates was reported in the same area by Escalera et al. (2019), with a mean total abundance of 1.0 × 10^6^ cells/L and a maximum of 2.9 × 10^6^ cells/L, two orders of magnitude higher than in TNB.

**Table 3:** Results of the pigments and chemotaxonomic analysis of the phytoplankton community of the sample stations.

Station	Area	Chl-a micro	Chl-a nano	Chl-a pico	Chl-c3	Chl-c2	Peridinin	Fucoxanthin	19hf	Diadinoxanthin	Alloxanthin	Diatoxanthin	Zeaxanthin	Lutein
ST12	TNB	1.29	0.49	0.73	0.14	0.25	0	0.48	0.37	0.07	0.01	0	0	0.01
ST14	TNB	NA	NA	0.15	0.05	0.47	0.05	1.43	0.05	0.18	0.03	0.01	0	0
ST15	TNB	1.06	1.22	0.11	0.03	0.26	0	0.85	0.04	0.15	0	0	0	0
ST18	TNB	1.33	0.97	0.09	0.03	0.26	0.04	0.82	0.05	0.14	0	0.01	0.01	0
ST19	TNB	NA	NA	NA	0.07	0.31	0	0.75	0.11	0.1	0	0.01	0	0
ST21	TNB	0.08	1.04	0.18	0.02	0.16	0	0.48	0.09	0.09	0	0	0	0
ST23	TNB	1.18	0.69	0.15	0.03	0.24	0	0.76	0.05	0.15	0.01	0.01	0.01	0.01
ST39	TNB	0.79	0.4	0.08	0.04	0.15	0	0.44	0.1	0.06	0.01	0.01	0.01	0
ST43	RSOW	NA	NA	0.08	0.03	0.36	0.09	1.05	0.05	0.17	0	0	0	0
ST44	RSOW	0.6	0.24	0.25	0.06	0.17	0	0.27	0.01	0.04	0	0	0	0.01
ST45	RSOW	0.13	0.49	0.12	0.05	0.12	0.02	0.15	0.01	0.05	0	0	0	0.01
ST46	RSOW	0.38	1.05	0.2	0.18	0.32	0	0.22	0.05	0.09	0	0.01	0	0.04
ST47	RSOW	0.41	0.55	0.26	0.12	0.02	0	0.15	0.03	0.01	0	0	0.01	0
ST48	RSOW	NA	NA	0.11	0.16	0.24	0	0.15	0.04	0.04	0	0	0	0.02
ST49	RSOW	1.33	0.86	0.19	0.12	0.31	0	0.49	0.01	0.07	0	0.02	0	0.01
ST51	RSOW	0.07	0.13	0.09	0.08	0.21	0	0.34	0.01	0.05	0	0.01	0	0.01
ST53	RSOW	0.89	0.07	0.18	0.1	0.32	0.04	0.53	0.27	0.07	0	0.01	0	0.01
ST54	RSOW	1.29	0.23	0.17	0.19	0.45	0	0.8	0.4	0.14	0	0.01	0	0.02
ST55	RSOW	1.87	0.18	0.17	0.17	0.39	0	0.53	0.59	0.13	0	0.01	0	0.02
ST59	RSOW	1.41	0.32	0.23	0.1	0.27	0	0.45	0.02	0.08	0	0.01	0	0.02

*Pigments concentration is expressed as μg/L. Chl-a micro, Chl-a nano, and Chl-a pico indicate the abundance of size classes micro-, nano-, and picophytoplankton in terms of biomass (μg of Chl-a/L). Chl-c2 – Chlorophyll C2; Chl-c2 – Chlorophyll C2; and 19hf – 19'-Hexanoyloxyfucoxanthin.*

**Table 4:** Maximum photochemical quantum yields of PS II (*F*v/*F*m) and percentage contribution of phytoplankton functional groups to the total phytoplankton biomass estimated by a chemotaxonomic method.

Station	Sampling area	*F*v/*F*m	Chlorophyta (%)	Cryptophyta (%)	Cyanophyta (%)	Diatom (%)	Dinophyta (%)	Haptophyta (%)	Prasphyta (%)
ST12	TNB	0.46	3.96	1.52	0	41.95	12.22	40.35	0
ST14	TNB	0.38	0.36	9.47	0.13	179.32	0.72	0	0.26
ST15	TNB	0.3	0.33	0.27	0.12	115.92	0.72	0	0.23
ST18	TNB	0.2	0.1	0.08	1.61	92.32	0	0	0.07
ST19	TNB	0.32	0.35	0.29	0.13	104.74	0	16.81	0.25
ST21	TNB	0.24	0.2	0.17	0.07	65.79	0	0	0.15
ST23	TNB	0.2	0.21	2.53	0.08	97.99	0.25	0	0.15
ST39	TNB	0.41	0.12	1.71	1.1	54.92	0	9.97	0.09
ST43	RSOW	0.23	0.16	0.13	0.06	120.87	0.01	0	0.11
ST44	RSOW	NA	4.19	0.1	0	44.3	0	16.93	0
ST45	RSOW	0.33	2.83	0.03	0	16.8	4.64	19.84	0
ST46	RSOW	0.3	12.28	0	0	18.47	0	80.73	0
ST47	RSOW	0.28	NA	NA	NA	NA	NA	NA	NA
ST48	RSOW	0.45	8.54	0	0	15.38	0	54.76	0
ST49	RSOW	0.42	4.36	0	0	49.24	0	46.15	0
ST51	RSOW	0.3	2.83	0.09	0	42.7	0	33.88	0
ST53	RSOW	0.26	2.84	0.04	0	58.23	0	26.14	0
ST54	RSOW	0.36	5.4	0	0	79.83	0	56.82	0
ST55	RSOW	0.3	7.15	0.05	0	57.25	0	44.01	0
ST59	RSOW	0.27	5.48	0.13	0	57.53	0	41.01	0

### Diversity of the Bacterial Community

**Figure 3:**
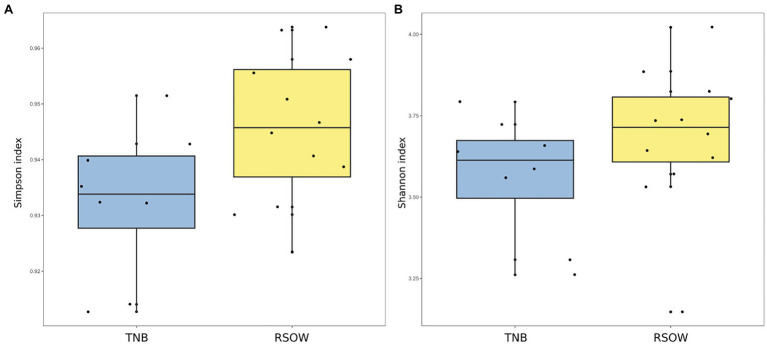
Alpha diversity metrics across the two sampled areas. **(A)** Simpson diversity index and **(B)** Shannon diversity index.

**Figure 4:**
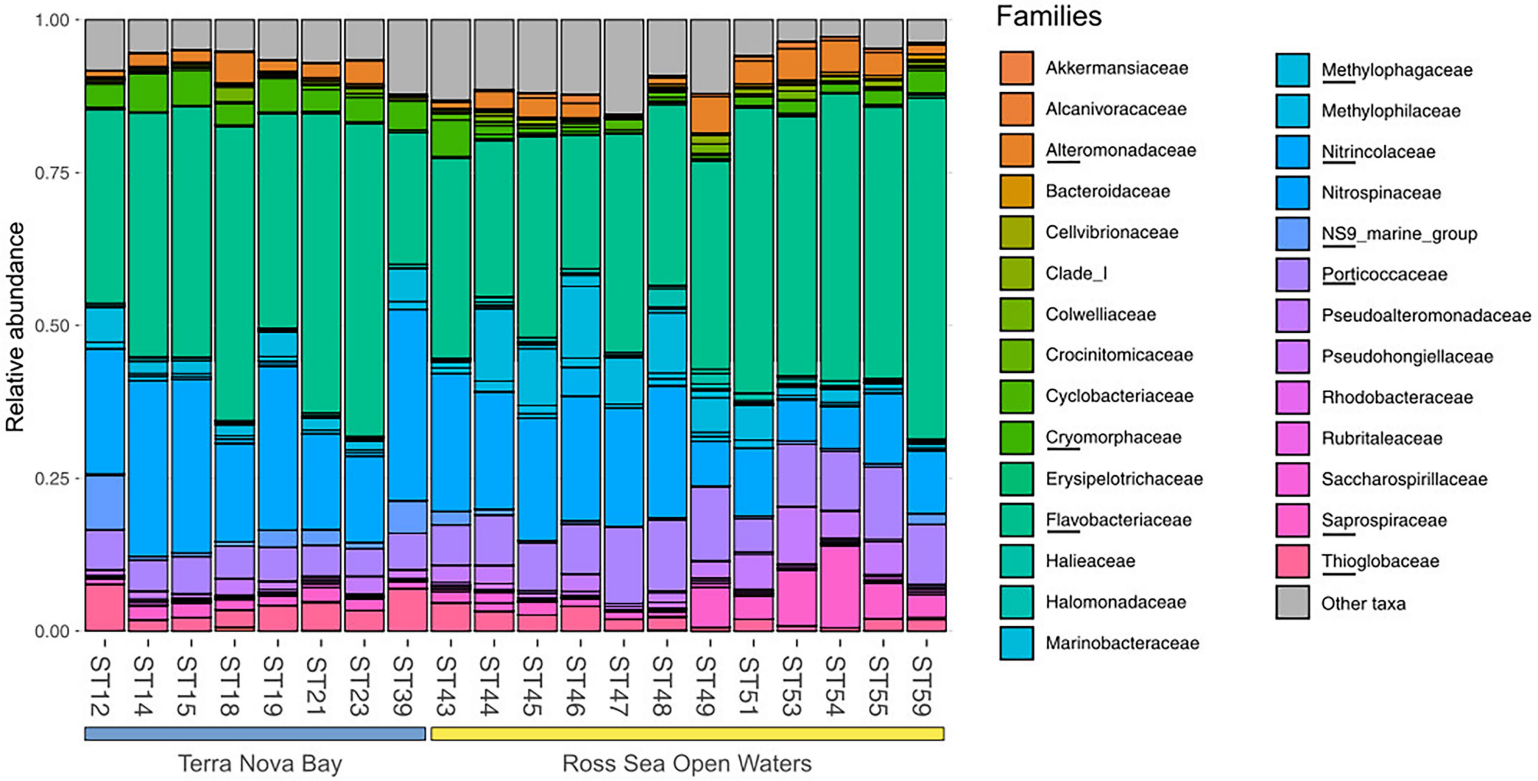
Family level distribution of the 16S rRNA diversity at the deep-chlorophyll maximum of the sampled stations. Only the most abundant families are reported, while the rare taxa are grouped together in the *Other taxa* category. Stations are grouped with a horizontal bar in two distinct clusters representing the TNB stations and the RSOW stations. Relative abundance reported as 1 = 100% of the total bacterial reads.

Bacterial diversity was investigated using the 16S rRNA gene sequence. After quality check and data filtering, a total of 535,009 reads were obtained and used to identify 703 unique ASVs. Simpson and Shannon diversity index showed higher diversity in the stations of RSOW than TNB (Figure 3), albeit the differences were not statistically significant (Kruskal–Wallis test). Despite these differences, the bacterial community structure at the phylum level was similar among the stations of the two areas. This apparent similarity is still visible all the way to the family level (Figure 4). Sequences belonging to the Bacteroidetes and Proteobacteria represented the most abundant phyla in all the samples, with on average 50.1% of the reads assigned to the Bacteroidetes and 48.4% to the Proteobacteria, respectively. In addition, sequences classified as belonging to the phyla Cyanobacteria, Firmicutes, Actinobacteria, and Verrucomicrobia were detected in almost all samples.

**Figure 5:**
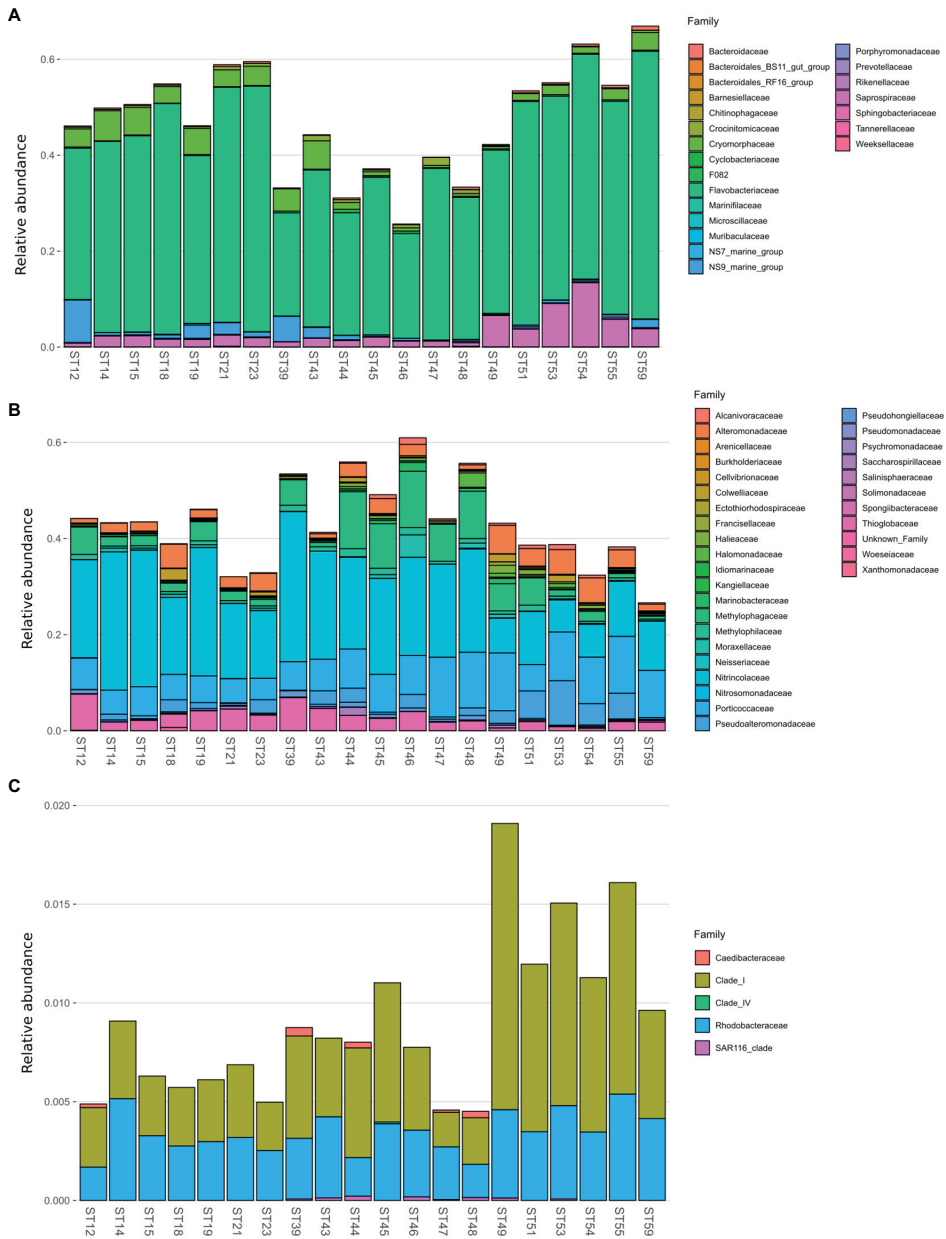
Family level distribution of the 16S rRNA diversity within the class *Bacteroidia* **(A)**, Gammaproteobacteria **(B)**, and Alphaproteobacteria **(C)** for the sample stations.

The phylum Bacteroidetes was dominated by the class of Bacteroidia, with the Flavobacteriales representing the most abundant order. Among Flavobacteriales, several ASVs were assigned to the genera *Polaribacter*, *Aurantivirga*, and *Brumimicrobium*, representing the top genera in the studied area (Figure 5A). Within the Proteobacteria, most sequences were affiliated to the class Gammaproteobacteria (47.41%), abundant both in the coastal (TNB) and in the offshore (RSOW) stations, followed by a lower percentage of ASVs assigned to the class Alphaproteobacteria (about 1% of total ASVs). Gammaproteobacteria were mainly represented by the orders Oceanospirillales, more abundant in the stations of the TNB area than in RSOW area (23.59% of ASVs found in TNB vs. 16.74% found in RSOW), followed by Cellvibrionales and Alteromonadales, which instead were more abundant in the offshore area (Figure 5B). Within the Oceanospirillales, several species were affiliated with the genera *Alcanivorax*, *Oleispira*, *Halomonas*, and *Profundimonas*, all known hydrocarbon degraders, distributed almost all the sampled stations, while among the Cellvibrionales, the main representatives were members of the clades SAR92 and OM60(NOR5). In addition, among Alteromonadales, we found mainly sequences classified as members of the *Pseudoalteromonas*, *Marinobacter*, and *Colweilla* genera. Alphaproteobacteria were mainly represented by the order SAR11_Clade and Rhodobacterales, uniformly distributed in both the studied areas representing on average 0.55 and 0.33% of the total reads, respectively (Figure 5C).

While the abundance of possible hydrocarbonoclastic bacteria (identified based on the similarity to known isolates) was relatively low, representing at most 3% of the total community (Supplementary Figure 1), they were distributed differentially among the sampled areas. Members of the genera *Alcanivorax*, *Marinobacter*, and *Oleispira* were present in all the sampled stations, with a higher abundance in the offshore stations of RSOW with, on average, 0.55, 0.46, and 0.29% of the total reads, respectively. Albeit with a lower abundance, we found also reads classified as *Methylophaga* and *Methylobacillus* in several stations of the RSOW area, while completely absent in the TNB stations.

To explore the bacterial community composition between the stations and to identify the environmental drivers responsible for its structuring, we investigated the beta diversity distribution using both abundance weighted and unweighted dissimilarity indices. The principal coordinate analysis (PCoA) using either the abundance weighted and unweighted Unifrac distance index shows a clear separation between the two studied areas in both plots (Supplementary Figures 2 A,B). Abundance weighted estimates of the beta diversity show a total of 72.5% of the variance visualized on the plot (Supplementary Figure 2 A), with the two study areas clearly separating along the PCoA axis 2 (explaining 21% of the variance). The only exception to this separation is represented by station ST43, which represents the first station of the RSOW area but is geographically located near the TNB stations (Figure 1). Unweighted diversity measures show a less pronounced separation among the two sampled areas, with a lower percentage of the variance accounted for by PCoA axis 1 and 2 (cumulatively 29% of the variance; Supplementary Figure 2 B). Direct comparison of the two plots revealed that the stations had on average similar species, and that differences were due variations in the abundant species.

**Figure 6:**
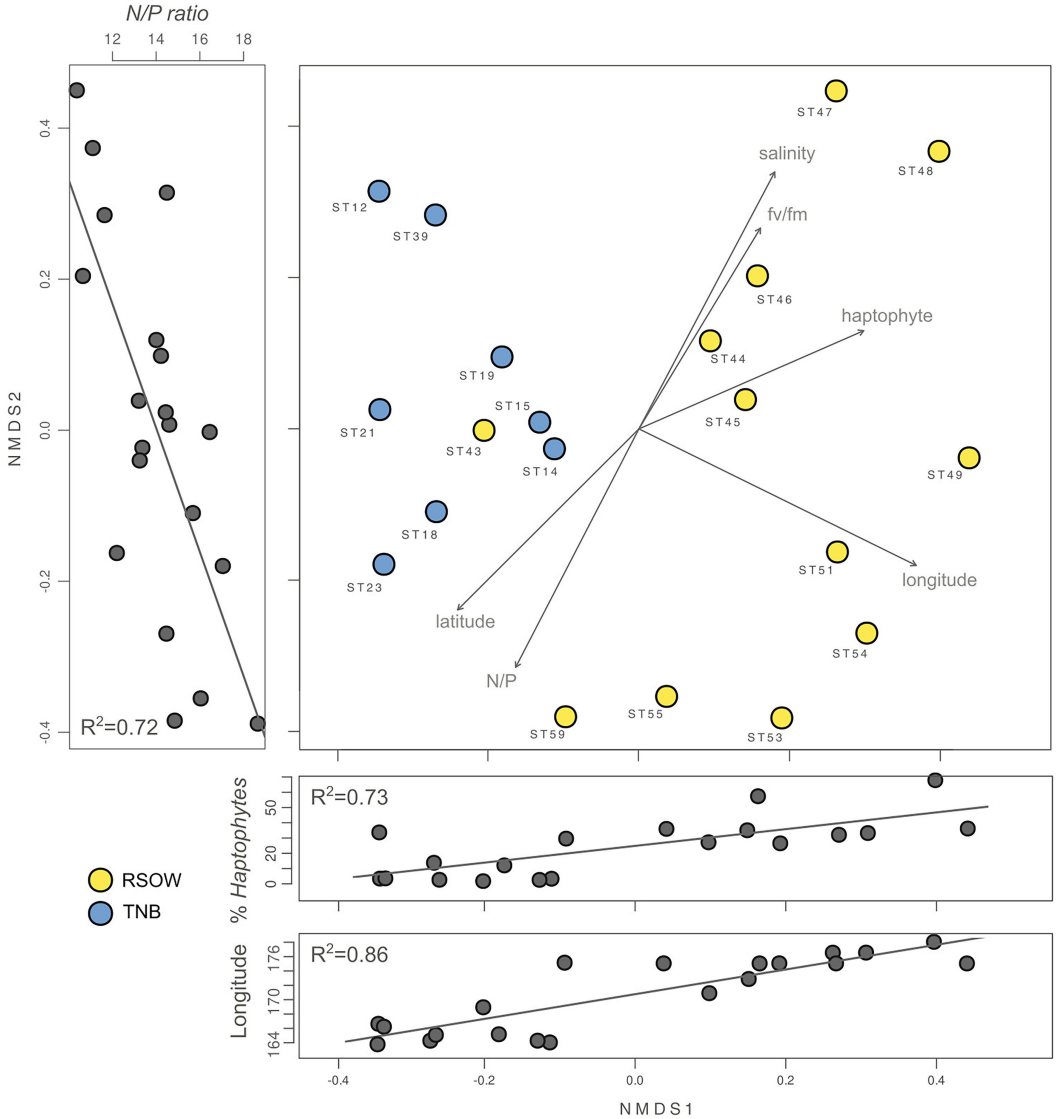
Non-metric multidimensional scaling (nMDS, stress 0.02) plot of the 16S rRNA gene amplicon microbial diversity based on Jaccard dissimilarity measure overlaid with environmental vector fitting. The lateral panels show the Pearson moment correlation (*R*^2^) between the respective nMDS axis and selected environmental and phytoplankton variables.

To investigate the role of measured environmental parameters and the phytoplankton community composition in driving the bacterial diversity in the two studied areas, we performed nMDS ordination based on the abundance weighted Jaccard dissimilarity measure followed by the environmental and community composition vector fitting (Figure 6). Similarly to the results obtained with the abundance weighted PCoA analysis, the nMDS showed a clear separation among the two areas with the exception of station ST43. Linear vector fitting against the nMDS ordination revealed that the main factors explaining the bacterial diversity were the geographic location (represented by the longitude of the sampled station) and the haptophytes relative abundance, with correlation coefficients of *R*^2^ = 0.86 and *R*^2^ = 0.72 against nMDS1, respectively (Figure 6; Table 5). The nitrogen to phosphorus ratio (N/P ratio), was instead strongly correlated with nMDS2 (*R*^2^ = 0.73), together with salinity and the maximum photosynthetic quantum yield (*F*v/*F*m), showing a correlation coefficient of *R*^2^ = 0.69 and *R*^2^ = 0.46, respectively.

**Table 5:** Results of the linear vector fitting of the environmental and phytoplankton community predictors on the bacterial diversity nMDS.

Factor	nMDS1	nMDS2	*r* ^2^	*adj.p*
Latitude	0.67728	−0.73573	0.5999	0.0059^**^
Longitude	0.23616	0.97171	0.7893	0.0004^***^
Depth	−0.48966	0.87191	0.1078	0.527
Salinity	−0.9096	0.41548	0.7535	0.0009^***^
Temperature	0.57759	−0.81633	0.1134	0.5381
Chl-a	0.54018	−0.84155	0.0538	0.7313
Chl-a micro	0.97747	−0.21105	0.1972	0.2993
Chl-a nano	−0.79477	−0.60691	0.1933	0.3013
Chl-a pico	−0.97243	−0.23321	0.0647	0.8328
Chl-c3	−0.14116	0.98999	0.3955	0.0699
Chl-c2	0.65972	0.75151	0.4361	0.0445^*^
Peridinina	0.96283	−0.27012	0.0451	0.7856
Fucoxantina	0.81665	−0.57713	0.3592	0.0897
19hf	0.9484	0.31707	0.0996	0.5752
Diadino	0.80687	−0.59073	0.2638	0.1863
Alloxan	−0.29111	−0.95669	0.3605	0.0841
Diatox	0.75075	0.66058	0.2646	0.1898
Zeaxant	0.27854	−0.96042	0.3408	0.0936
Luteine	−0.32573	0.94546	0.2734	0.1695
*F*v/*F*m	−0.81871	0.57421	0.4581	0.0381^*^
Chloro	−0.34356	0.93913	0.3317	0.1016
Crypto	−0.19376	−0.98105	0.4197	0.0382^*^
Cyano	−0.09494	−0.99548	0.2406	0.2215
Diato	0.72274	−0.69112	0.4541	0.0362^*^
Dino	−0.98792	−0.15499	0.2126	0.2495
Hapto	−0.32373	0.94615	0.5007	0.0233^*^
Pras	0.14949	−0.98876	0.4227	0.0453^*^

* adj.p < 0.05;

** adj.p < 0.01;

*** adj.p < 0.001.

**Figure 7:**
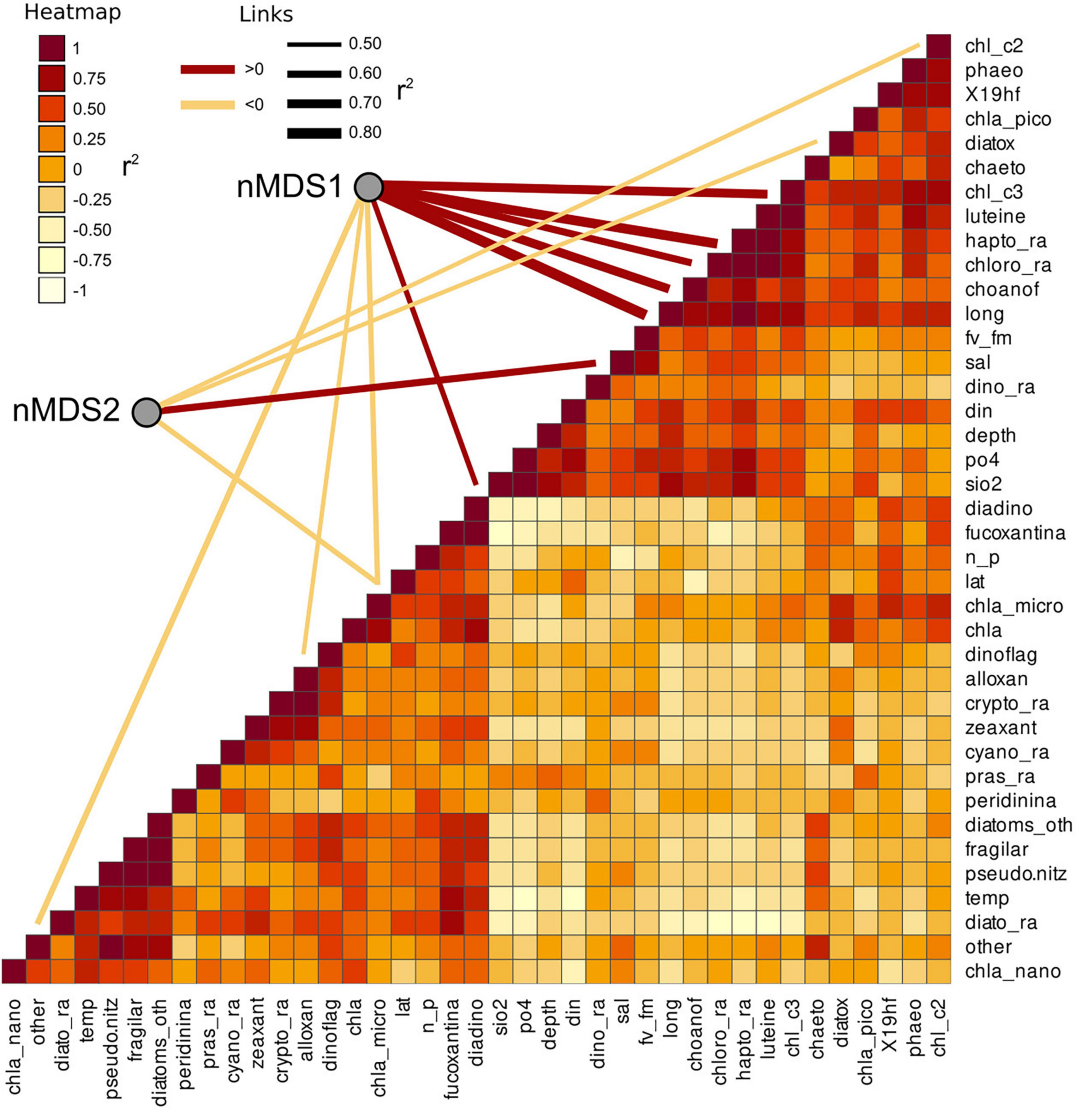
Collinearity of the environmental and phytoplankton variables used as predictors of bacterial beta diversity. The nMDS axes are connected with significant predictors through a line showing the correlation direction (color) and intensity (line thickness). Collinearity among the predictors was calculated as Pearson moment correlation and plotted as a heatmap.

Several other variables were identified by the linear vector fitting as significatively correlated with nMDS axis 1 and 2. This was likely due to the high degree of collinearity present among the environmental parameters and among the phytoplankton composition descriptors (Figure 7). Collinearity was investigated using Pearson moment correlation among the predictors used for the linear vector fitting, together with the results of the vector fitting for both nMDS axis (Figure 7; Table 5).

### Differential Distribution of Key Bacterial Genera in the Ross Sea Surface Waters

**Figure 8:**
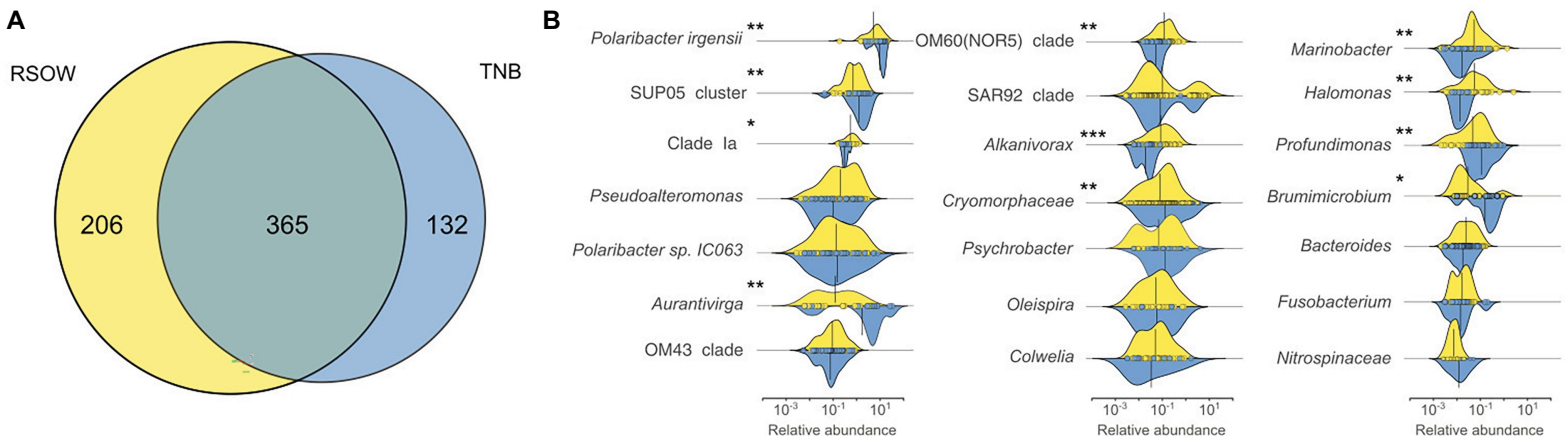
Shared and unique Amplicon Sequence Variants (ASVs) among the two sampled areas **(A)** and density function of the abundance of the top genera shared among the sampled areas **(B)**. Density plots showing the differential distribution of the major genera between the two sampled areas. The *x* axis represents the relative abundance of each ASV in the genera while the *y* axis shows the relative density functions. The vertical solid line represents the mean relative abundance for those genera in the area. Differentially abundant genera are marked with an * according to the result of the Kruskal–Wallis test (^*^adj.p < 0.05; ^**^adj.p < 0.01; and ^***^adj.p < 0.001).

The overall distribution of the bacterial community in the multivariate ordination shows that the two areas are different in term of community assemblages and that the separation between the two sampled areas is driven by abundant species. We determined unique and shared ASVs between TNB and RSOW area (Figure 8A), revealing that the shared community is composed of 365 ASVs, while 132 and 206 unique ASVs are found in TBN and RSOW samples, respectively. We identified the top bacterial genera shared among the two studied areas (Figure 8B), and identified those showing a differential distribution. The results show several well-known members of the Antarctic bacterioplankton community as the most abundant genera, some of which are differentially distributed among the two areas. Sequences belonging to the *Polaribacter* genus were highly abundant in the dataset, representing on average 47.1% of the total reads. Among them, 41.1% were related to *Polaribacter irgensii* (Table 6), a psychrophilic heterotrophic gas vacuolate bacterium of the *Bacteroidetes* phylum, previously isolated from Arctic and Antarctic sea ice (Gosink et al., 1998). As shown in Figure 8B, reads classified as *P. irgensii* are differentially distributed with a higher percentage of read found in the coastal area of TNB with respect to the offshore (24.3% in TNB vs. 16.8% in RSOW, *adj.p* < 0.01). The remaining reads are classified as members of *Polaribacter* sp. IC063 are instead uniformly distributed among the sampled areas. Members of the SUP05_cluster, a bacterial taxon comprising chemolithoautotrophs generally detected in fluids and hydrothermal plumes (Sunamura et al., 2004; Sunamura and Yanagawa, 2015) or in low oxygen areas of the water column (Walsh et al., 2009), follow a trend similar to *P. irgensii*, with the percentage of ASVs assigned to this taxon higher in the coastal area of TNB with respect to the offshore RSOW stations (4.1 vs. 2.1%, respectively). Although with a lower abundance (less than 1% of the total reads), we found that the ASVs associated to the genera *Profundimonas* and *Brumicrobium*, cold-adapted and facultatively anaerobic heterotrophic bacteria generally found in marine environments (Bowman et al., 2003; Margesin, 2017), are also more prevalent in the TNB samples.

**Table 6:** Most abundant shared genera among the two sampled areas.

			Ez-Biocloud identification		
Genus (Silva)	*χ* ^2^	*adj.p*	Closest relative	% Similarity	Acc. no.
*Aurantivirga*	7.7282	0.0054^**^	*Aurantivirga* sp. ARTD_S	99.35	ARTD01000013
*Polaribacter*	0.4190	0.5174	*Polaribacter* sp. IC063	98.71	U85885
*Polaribacter_1*	9.4464	0.0021^**^	*Polaribacter irgensii*	98.71	U73726
SAR92 clade	0.1087	0.7416	SAR92 clade AY386340_s	98.13	AY386340
SUP05 cluster	6.1877	0.0129^*^	*Thioglobus* sp. CDSC_s	98.65	CDSC02000433
*Pseudoalteromonas*	2.3486	0.1254	*Pseudoalteromonas marina*	99.38	AY563031
OM43 clade	0.0998	0.7521	*Methylophilaceae*; OM43_g	100	HQ672174
Clade Ia	4.3393	0.0372^*^	*Pelagibacter ubique*	100	CP000084
*Psychrobacter*	0.1015	0.7500	*Psychrobacter namhaensis*	100	AY722805
*Profundimonas*	8.7708	0.0031^**^	*Thalassotalea crassostreae*	95.63	CP017689
*Marinobacter*	7.8064	0.0052^**^	*Marinobacter salarius*	100	CP007152
*Brumimicrobium*	4.3734	0.0365	*Brumimicrobium glaciale*	98.06	AF521195
*Halomonas*	7.2385	0.0071^**^	*Halomonas gomseomensis*	98.38	AM229314
*Colwellia*	0.5797	0.4464	*Thalassotalea* sp. JN018842_s	95.63	JN018842
*Oleispira*	0.0810	0.7759	*Oleispira lenta*	100	EU980447
OM60(NOR5) clade	7.1700	0.0074^**^	*Marimicrobium* sp. FJ717233_s	96.88	FJ717233
*Alcanivorax*	13.0233	0.0003^***^	*Alcanivorax* sp. BDAS-s	98.75	BDAS01000027
*Bacteroides*	0.2139	0.6437	*Bacteroides dorei*	100	ABWZ01000093
*Fusobacterium*	0.0416	0.8384	*Fusobacterium* sp. EU772717_s	100	EU772717
*Cryomorphaceae*	6.3006	0.0121^*^	*Cryomorphaceae*; FQ032815_g	100	HQ730063
*Nitrospinaceae*	4.3924	0.0361^*^	*Nitrospina* sp. EU035855_s	100	EU035855

The table reports the results of the statistical test for differential distribution and the information relative to the closest cultured relative identified using the Ez-Biocloud identification software.

* adj.p < 0.05;

** adj.p < 0.01;

*** adj.p < 0.001.

An opposite trend was followed by the reads classified as members of the genera *Aurantivirga*, and, with a markedly lower abundance, by the reads affiliated to Clade Ia, OM60 (NOR5) clade, *Alcanivorax*, *Marinobacter*, and *Halomonas* (Figure 8B). *Aurantivirga* is a member of *Flavobacteriaceae* whose type strain, *Aurantivirga profunda*, has been isolated in the deep-sea waters of the Pacific Ocean (Song et al., 2015). Our data show that the ASVs belonging to *Aurantivirga* are abundant in the RSOW area (on average 4.7%) and become substantially lower in the coastal area of TNB (0.7%, *adj.p* < 0.01). Our results show that the ASVs related to Clade Ia are significantly more abundant in the RSOW area (0.7%) with respect to the TNB area (0.3%). A similar distribution was found for members of the OM60 (NOR5) clade with a higher abundance in the offshore stations with respect to the coastal stations (0.6% in RSOW vs. 0.2% in TNB). The genera containing obligate or facultative hydrocarbon oxidizers *Alcanivorax*, *Oleispira*, and *Marinobacter* were also differentially distributed among the sampled areas, showing statistically significant higher abundances in the stations of the RSOW compared to TNB (*adj.p* < 0.001 for *Alcanivorax* and *adj.p* < 0.01 for *Oleispira* and *Marinobacter*).

**Figure 9:**
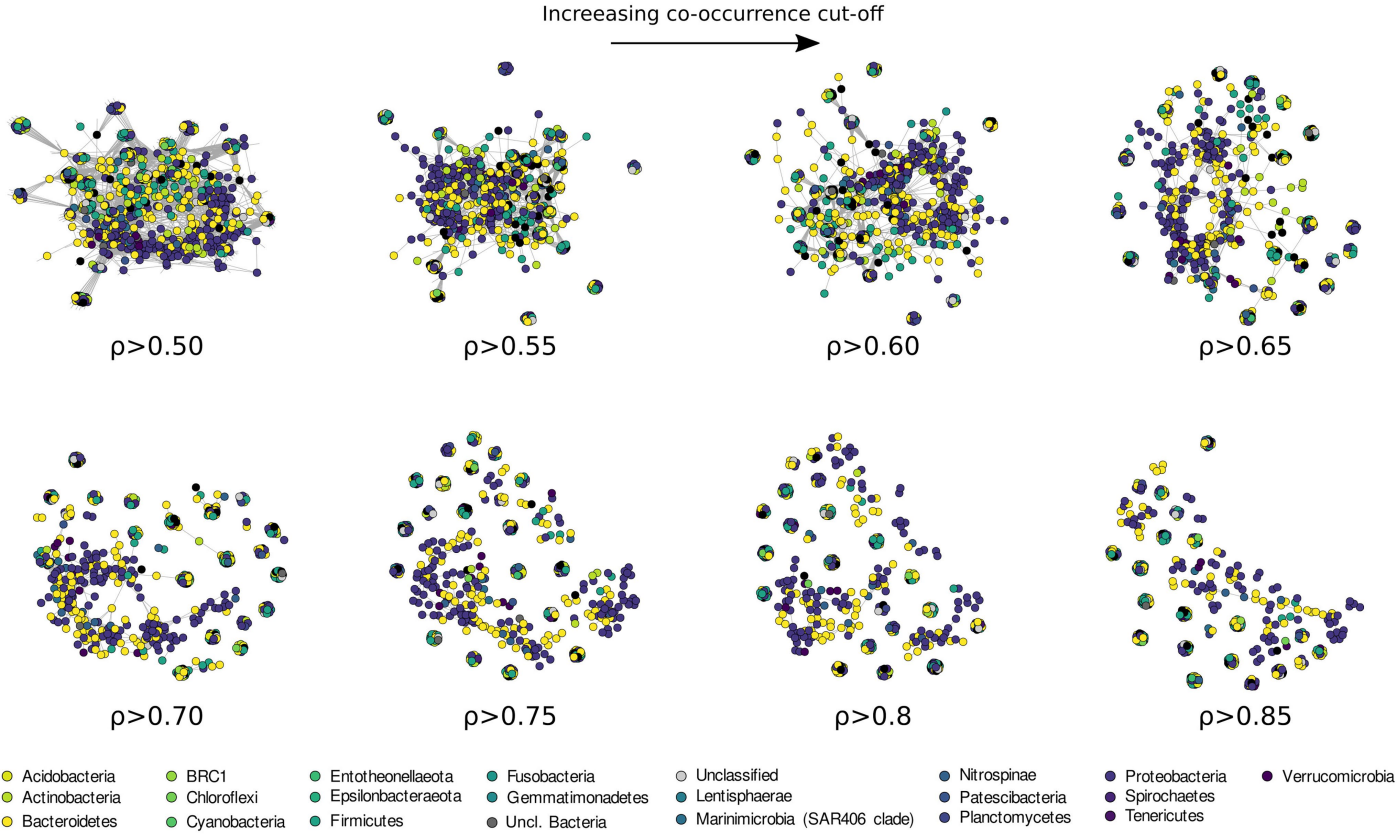
Co-occurrence network analysis drawn with increasing Spearman correlation cut off and colored according to the phyla classification of each ASVs.

Patterns of co-occurrence among the more prevalent ASVs (here defined as occurring in at least 20% of the sampled stations) were investigated using different cut-off levels of Spearman correlation coefficient, between 0.5 and 0.85 (Figure 9; Supplementary Table 1). The results revealed a phylogenetically heterogeneous cluster of highly co-occurring ASVs present at low threshold of correlation (from 0.5 to 0.65 ρ), progressively breaking down into small (20–30 ASVs) phylogenetically heterogeneous highly correlated clusters.

## Discussion

The Ross Sea is a complex mosaic of subsystems with physical, chemical, and biological features that respond to biotic and abiotic forcing at different temporal and spatial scales (Smith et al., 2007, 2014; Mangoni et al., 2017; Bolinesi et al., 2020b). Although the phytoplankton blooms dynamics in the Ross Sea have been well described, the drivers regulating the temporal and spatial distribution of the blooms are still debated (Arrigo et al., 1999; DiTullio et al., 2000; Mangoni et al., 2017, 2019; Saggiomo et al., 2021).

Among the biotic processes structuring the phytoplankton community, the interactions with the bacterioplankton has been proposed to be an important factor (Richert et al., 2019). For example, vitamin B12 availability, which is produced exclusively by bacterioplankton, has been suggested to be one of the drivers involved in the diatoms-Phaeocystis shift in the Ross Sea waters (Bertrand et al., 2007, 2015). Despite the bacterioplankton composition in the Ross Sea has been previously investigated (Lo Giudice et al., 2012; Silvi et al., 2016), the phytoplankton-bacterioplankton co-occurrence in the area has been poorly characterized. Many studies have suggested the existence of sophisticated ecological interactions between phytoplankton and bacteria in driving marine biological processes, which can range from a mutualistic exchange of biomolecules and nutrients to the competition for the same limiting inorganic nutrients (Amin et al., 2012, 2015; Seymour et al., 2017). In this study, we have investigated the bacterial diversity of the Ross Sea in relation to phytoplankton community structure in 20 stations located between the coastal area of TNB and the offshore waters of the central Ross Sea (RSOW). The two areas were characterized by different amounts of phytoplankton biomass and dominant functional groups (Figure 2), with highest levels of biomass near the coastline. *Pseudo-nitzschia* spp. and *Fragilariopsis* spp. were the most abundant species in TNB, while *P. antarctica* dominated the stations in the RSOW.

The bacterioplankton community was representative of Antarctic surface waters in accordance with previous studies (Abell and Bowman, 2005; Ghiglione and Murray, 2012; Grzymski et al., 2012; Wilkins et al., 2013), with Bacteroidetes and Proteobacteria representing the most abundant phyla. Among Bacteroidetes, members of the *Flavobacteriaceae*, commonly found bacteria in polar environments (Abell and Bowman, 2005; Williams et al., 2012), dominated both the sampled areas. These bacteria are generally classified as the key degraders of phytoplankton derived organic matter (Teeling et al., 2012; Buchan et al., 2014). Their presence in all the sampled stations suggests that these bacteria might play an important role in the organic carbon cycle of the Ross Sea, potentially impacting organic carbon transfer to higher trophic levels (Azam and Malfatti, 2007). Despite the apparent similarity in the community composition between the two areas (Figure 4), several differences were present at the Genus and ASVs level. Within our dataset, members related to the *Polaribacter* genus were dominant in all the sampled stations, and showed a statistically higher abundance in the TNB area (Figure 8B). The closest relative to our sequences was *P. irgensii*, with an average similarity of 98.7% (Table 6), a known psychrophilic heterotrophic marine bacterium. Previous studies have reported that *Polaribacter* species thrive during diatom-blooms (Teeling et al., 2012; Williams et al., 2012), potentially suggesting that their presence in the TNB area might be linked with the higher presence of diatom species we have identified. Similarly, a recent analysis revealed that species of the *Aurantivirga* genus are among the first prokaryotic taxa responding to diatom bloom in the Southern Ocean (Liu et al., 2020). Sequences related to the genus *Aurantivirga* are highly abundant in our TNB stations, suggesting once again a possible relationship with the high abundance of diatoms (Figures 2, 8B). Among the Proteobacteria, sequences related to the Gammaproteobacteria were detected in all the sampled stations, with a differential distribution of members related to the *Alteromonadales*, *Cellvibrionales*, and *Oceanospirillales* orders among the two studied areas. Within the *Alteromonadales*, sequences related to *Pseudoalteromonas*, *Marinobacter*, and *Colweilla* genera were dominant and in higher abundance in the RSOW stations (Figure 8B). These bacterial genera comprise cold-adapted marine bacteria generally detected in the Southern Ocean waters able to degrade simple sugar, amino acids, organic compounds, and hydrocarbons (Médigue et al., 2005; Methé et al., 2005; Singer et al., 2011; Rosenberg, 2013). The abundance of ASVs belonging to these psychrophilic bacterial groups in the offshore area could be explained by specific adaptation to the conditions found in offshore stations. Similarly, members of the *Cellvibrionales* order dominated the offshore area. These bacteria, generally affiliated to the Oligotrophic Marine Gammaproteobacteria (OMG) cluster (Cho and Giovannoni, 2004), are able to adapt to nutrient depletion and carbon limitation with the potential to harvest light for mixotrophic growth (Stingl et al., 2007; Spring and Riedel, 2013; Courties et al., 2014). We also detected sequences related to the clades SAR92 and OM60 (NOR5). Previous reports indicate that members of SAR92 clade dominate during the phytoplankton bloom in the Southern Ocean (West et al., 2008) and are able to establish a close relationship with the productive *P. antarctica* during the austral summer in the Amundsen sea polynya, suggesting an important role of this bacterial taxa during bloom formation and bloom longevity (Delmont et al., 2014). Consistent with this data, our results show a dominance of the SAR92 clade in the RSOW area which is dominated by *P. antarctica*. The ability to exploit different metabolic pathways based on the conditions found in the sea water, suggest that both *Alteromonadales* and *Cellvibrionales* can play an important role in the microbial food web, contributing to the functioning of the Antarctic marine ecosystems. Taken together, these observations suggest a tight coupling between the phytoplankton community structure and the dominant bacteria identified in the Ross Sea surface waters.

Among the top genera identified in our study, members belonging to the SAR11 order of the Alphaproteobacteria Clade Ia, were comparatively low in relative total abundance. Our results show that ASVs classified in this group were more abundant in the RSOW area. Species belonging to the SAR11 order comprise aerobic and free-living oligotrophic chemoheterotrophic bacteria globally distributed in marine environments (Morris et al., 2002). They are believed to contribute significantly to the carbon, nitrogen, and sulfur cycling in the Ocean (Malmstrom et al., 2004), and have been previously reported worldwide (Field et al., 1997; Morris et al., 2002; Brown et al., 2012). Members of the SAR11 group have been shown to be more abundant in the Subantarctic and polar fronts compared to Antarctic zones (Wilkins et al., 2013 and references therein). The low global abundance found in our dataset might be due to the lack of competitive advantage of SAR11 members in the presence of high molecular weight organic carbon during phytoplankton blooms. Members of Clade Ia identified in our dataset represent a specific subgroup of the order generally reported in cold waters (Brown et al., 2012; Delmont et al., 2019). The closest relative to our sequences was *Pelagibacter ubique*, the most common heterotrophic bacteria found in the ocean (Giovannoni, 1990; Giovannoni et al., 2005), with an average similarity of 100% (Table 6). Studies based on genome sequences analysis and *in situ* hybridization, revealed that *P*. *ubique* is an oligotrophic bacterium with a small genome size and a high metabolic activity, able to assimilate either dissolved free amino acids and dimethylsulfoniopropionate (DMSP; Malmstrom et al., 2004; Giovannoni and Stingl, 2005). DMSP is an organosulfur compound produced by several phytoplankton cells, which can perform a double function in polar microalgae, as osmolyte or cryoprotective agent (Kirst et al., 1991; Karsten et al., 1996). Several reports indicate that *P*. *antarctica* is one of the leading producers of DMSP in the Ross Sea (DiTullio and Smith, 1995; Ditullio et al., 2003).

Interestingly, our dataset reveals the presence of obligate or facultative hydrocarbon oxidizers in the Ross Sea surface waters, albeit at abundances below ~3%. While the presence of facultative hydrocarbon oxidizers *per se* is not indicative of hydrocarbon contamination in the environment, our data reveal that the obligate hydrocarbonoclastic genera *Alcanivorax* and *Oleispira* (Yakimov et al., 1998, 2003) were present in all the sampled stations, and markedly more abundant in the RSOW stations. *Alcanivorax* and *Oleispira* are two bacterial genera found globally in extremely low abundances (Cafaro et al., 2013), but can become transiently dominant, with relative abundances up to 70–90% of prokaryotic cells, in the presence of hydrocarbon spills (Kasai et al., 2002a,b). Their presence in our dataset is interesting and can be explained in several different ways. While their abundance is significantly lower than reported after oil spillage events (Kasai et al., 2002b; Harayama et al., 2004), it is possible that hydrocarbon and exhaust oils released by the large number of ships transiting the Ross Sea every summer are responsible for keeping them above the normal background levels. Antarctic tourism has been steadily increasing over the years, with over half a million tourist landings reported for the 2017–2018 season (Palmowski, 2020), and fishing activities in proximity of the productive Antarctic waters have also increased (Brooks and Ainley, 2017). Alternatively, previous studies have proposed that members of the *Alcanivorax* genera are able to maintain viable status in uncontaminated marine waters degrading natural lipids of bacterial and phytoplankton origin, released in the water column due to exudation, sloppy feeding, or viral lysis (McGenity et al., 2012; Lea-Smith et al., 2015; Zadjelovic et al., 2020). During our sampling, the phytoplankton community was undergoing a summer bloom, and thus all the mentioned processes, e.g., exudates, sloppy feeding by the zooplankton grazers, and viral induced lysis, might have contributed in increasing lipid concentrations in seawater. In addition to *Alcanivorax* and *Oleispira*, the facultative hydrocarbon degraders *Marinobacter* and *Halomonas* have been also identified (Supplementary Figure 1). Members of the genus *Marinobacter* are slightly or moderately halophilic, able to degrade both aliphatic and aromatic hydrocarbons. *Marinobacter* spp. capable of growing on hydrocarbons as the sole carbon source has been previously isolated from sediments (Gauthier et al., 1992). The genus *Halomonas* comprises marine halophilic and/or halotolerant bacteria, known to produce large quantity of exopolysaccharides (EPS) with rheological and active-surface properties (Calvo et al., 1998, 2002; Martínez-Checa et al., 2002; Pepi et al., 2005; Gutierrez et al., 2020). It is possible that *Halomonas*-producing EPS provides a mechanism to increase the bioavailability of hydrophobic compounds (e.g., hydrocarbons and lipid aggregates) that *Alcanivorax*, *Oleispira*, and *Marinobacter* utilize for growth.

Information regarding the structure of bacterial communities can be also investigated through the use of network analysis. Most microbial network analysis uses a single correlation threshold to identify meaningful interactions from a correlation matrix (Barberán et al., 2012; Williams et al., 2014; Giovannelli et al., 2016; Connor et al., 2017). This approach, while might provide useful information regarding the biological interactions in the community, requires *a priori* justification or a sensitivity analysis to demonstrate the robustness of the conclusion with respect to the selected threshold. In addition, single threshold co-correlation analysis is considered controversial by some authors (Hirano and Takemoto, 2019; Blanchet et al., 2020) and it is believed to increase the number of detected false positives. An alternative approach to identifying biological interactions is using co-occurrence to identify ecological response to common environmental factors, an approach recently applied with success across broad ecological gradients (Delgado-Baquerizo et al., 2018; Fan et al., 2018; Fullerton et al., 2021). Microbial co-occurrence networks can be also used to investigate community assembly and dynamics. To this end, we used an increasing correlation threshold to identify the structuring of the bacterioplankton community in the surface waters of the Ross Sea, preserving more ecological signal compared to a fix-threshold approach (Figure 9). With this approach, the degree at which the network breaks apart and changes in structure during thresholding can be used to identify processes influencing community assembly (Connor et al., 2017). As the threshold is increased, the largest network component becomes sparser as edges among nodes are removed (Figure 9). If modularity and other network statistics follow a linear trend during thresholding, this suggests the presence of a dominant core microbiome, often proposed to be critical or keystone components of the community (Faust and Raes, 2012; Mandakovic et al., 2018). Conversely, non-linear trends in network modularity suggest the absence of representative core microbiome and possibly the absence of keystone species.

Our data show that the bacterioplankton community is composed of phylogenetically heterogeneous clusters of bacteria showing progressively lower co-occurrence as the correlation threshold is raised (Figure 9). Usually, high levels of co-occurrence in microbial networks are interpreted as increasing biotic interactions among species, including competition for resources (Faust and Raes, 2012; Widder et al., 2014). The resulting sparse network we have obtained at higher level of correlation threshold (>0.60 ρ, Figure 9; Supplementary Figure 3; Supplementary Table 1), suggests that stochastic processes (e.g., neutrality, dispersion, and physical stress, see Holyoak et al., 2005), rather than competition, are responsible for community assemblage in the surface waters of the Ross Sea. Additionally, our results suggest the absence of a defined core microbiome in the studied area. This might be connected to different bacterial assemblages being associated to distinct phytoplanktonic communities identified in the TNB and the RSOW areas. Despite this, the presence of small clusters (20–30 ASVs) of phylogenetically diverse but recurrent ASVs suggests a strong functional redundancy among the most representative bacterial species. Ecological network theory predicts that communities of tightly connected species should be more fragile. Our data suggest that the higher fragmentation identified might be the dynamic response of the bacterial community to phytoplankton related dynamics, which continuously rework and redistribute microbial niches as blooms succession progresses (Luria et al., 2017).

## Conclusion

The role of bacterioplankton in Antarctic waters has been reevaluated in the last decades, highlighting the importance of the bacterioplankton-phytoplankton interaction in the ocean carbon cycling, biogeochemical processes, and trophic food chain. Data from our study indicate that bacterioplankton diversity was clearly linked to the phytoplankton community structure and distribution in the Ross Sea surface waters in the austral summer 2017. Members of the Bacteroidetes and Proteobacteria phyla were abundant in all the sampled stations, with a dominance of heterotrophic bacterial species (i.e., *Polaribacter* genus) in presence of diatom blooms in the coastal area of TNB, and a dominance of oligothrophic and mixotrophic bacterial species in presence of haptophytes blooms in the offshore area. The overall picture emerging suggests the existence of specific rather than random interactions between dominant phytoplankton-bacterioplankton species that contributes to the organic matter cycling in the Southern Ocean.

## Data Availability Statement

The datasets presented in this study can be found in online repositories. The names of the repository/repositories and accession number(s) can be found in the article/Supplementary Material.

## Author Contributions

OM, AC, and PR designed the study. MM, AC, PR, MSa, FB, and OM performed laboratory analyses. MM, AC, DG, MSe, MB, RM, and GD’E performed bioinformatics and statistical analyses. All authors contributed equally to the manuscript writing and revision.

## Funding

Samples were collected in the framework of Plankton biodiversity and functioning of the Ross Sea ecosystems in a changing Southern Ocean [P-ROSE – (PNRA16_00239)], and CDW Effects on glacial mElting and on Bulk of Fe in the Western Ross sea [CELEBeR – (PNRA16_00207)] projects – Italian National Antarctic Program – funded by the Ministry of Education, University and Research (MIUR), awarded to OM and PR, respectively. MM was supported by an Earth-Life Science Institute (Tokyo, Japan) visiting fellowship. This work was partially supported by the European Research Council (ERC) under the European Union’s Horizon 2020 research and innovation programme (grant agreement No. 948972) to DG.

## Conflict of Interest

The authors declare that the research was conducted in the absence of any commercial or financial relationships that could be construed as a potential conflict of interest.

## Publisher’s Note

All claims expressed in this article are solely those of the authors and do not necessarily represent those of their affiliated organizations, or those of the publisher, the editors and the reviewers. Any product that may be evaluated in this article, or claim that may be made by its manufacturer, is not guaranteed or endorsed by the publisher.

## Footnotes

^1^ www.genprobio.com
