# Supplementary material for: Bacterioplankton Diversity and Distribution in Relation to Phytoplankton Community Structure in the Ross Sea Surface Waters
Source: Front Microbiol. 2022 Jan 27;13:722900. doi: 10.3389/fmicb.2022.722900 (PMC8828583; doi:10.3389/fmicb.2022.722900)
Supplement: Supplementary file 1 [file Data_Sheet_1.pdf]

Supplementary Material

1 Supplementary Figures and Tables

1.1 Supplementary Figures

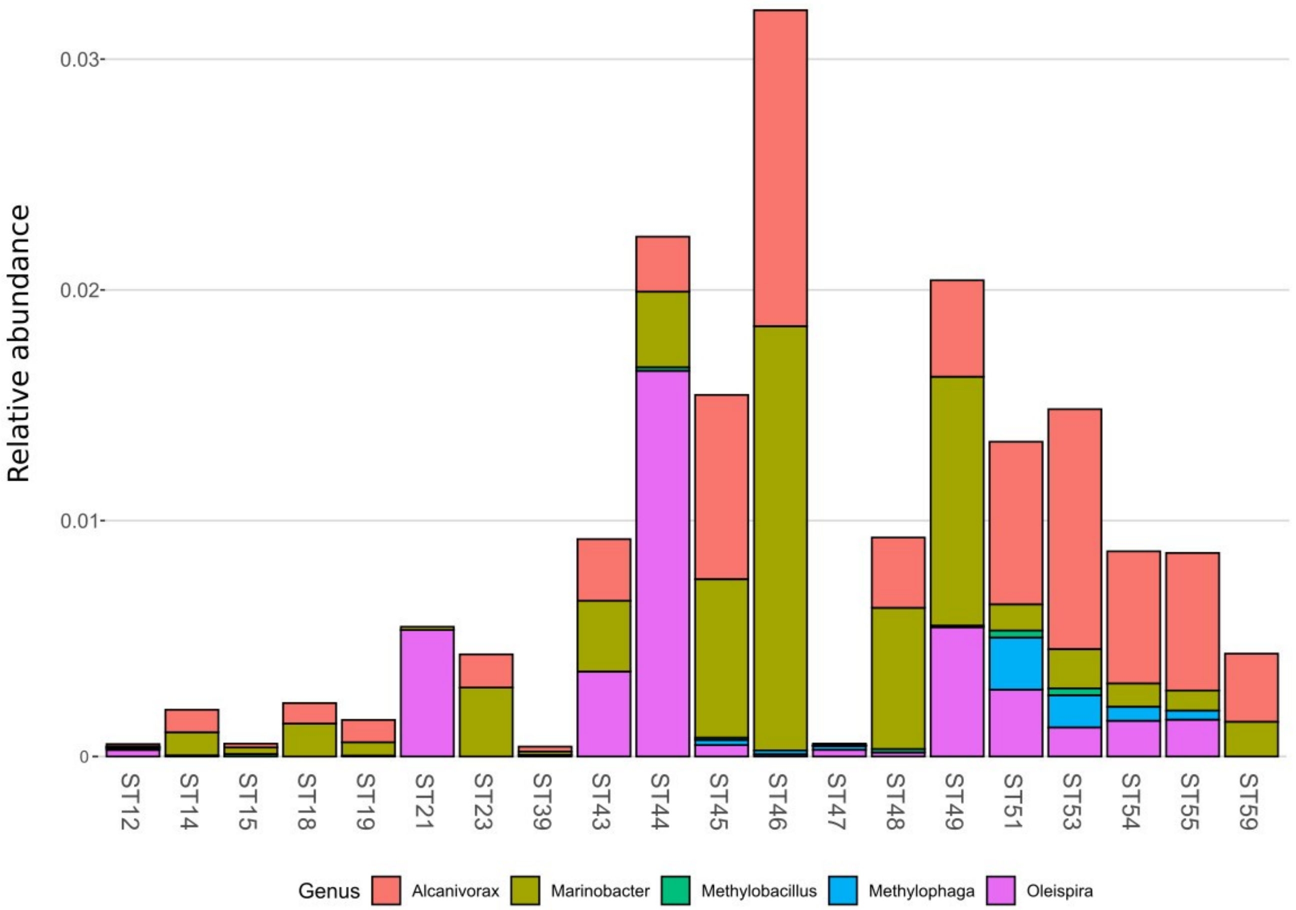

Supplementary Figure 1. Genus level distribution 16S rRNA diversity of the putative hydrocarbon degraders detected in the sampled stations.

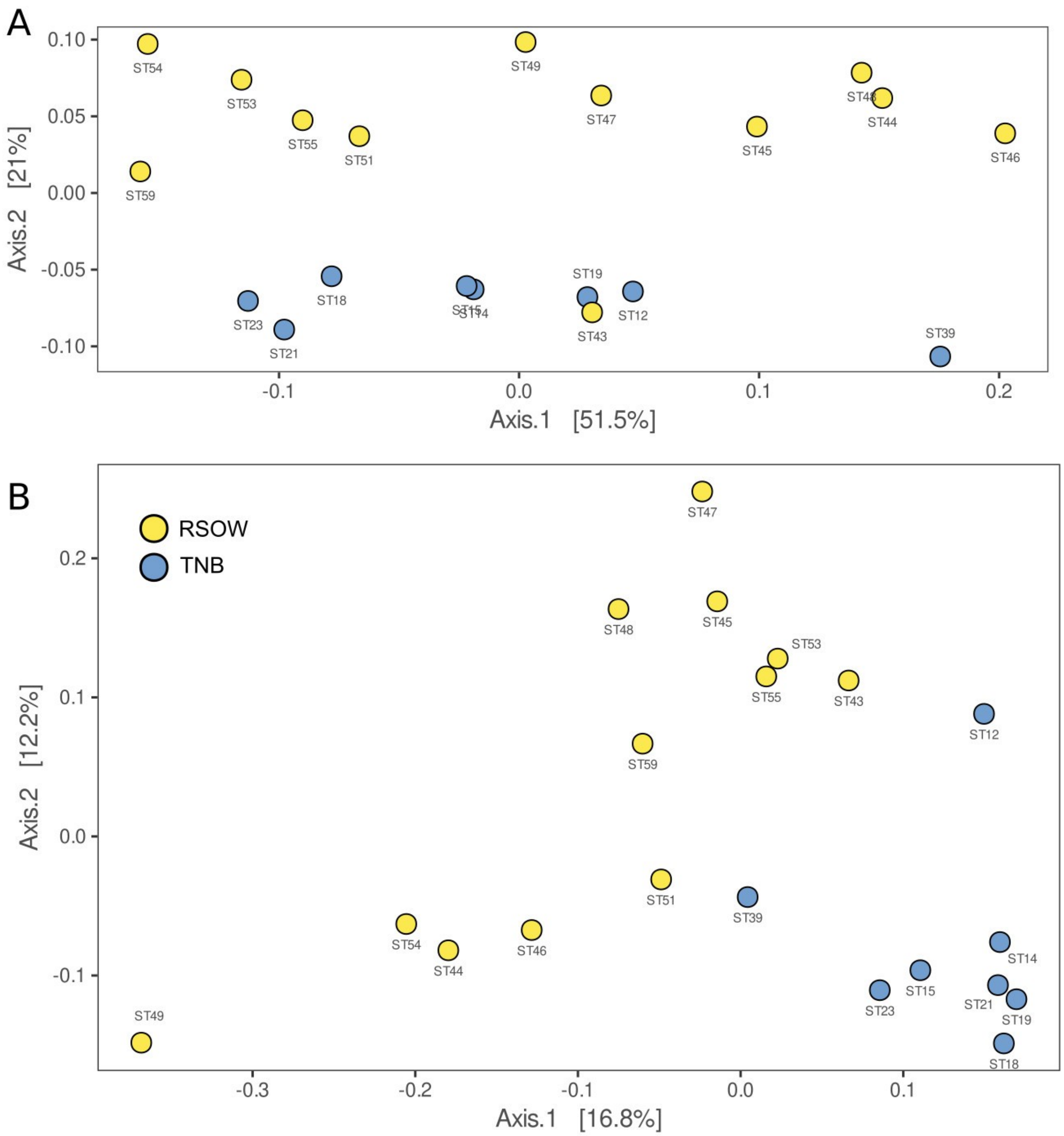

Supplementary Figure 2. Principal coordinate analysis (PCoA) plot of the 16S rRNA gene microbial diversity based on Unifrac dissimilarity measure in the sampled stations: A) abundance weighted; and B) unweighted PCoA plots. The amount of variance explained by each axis is reported within square brackets.

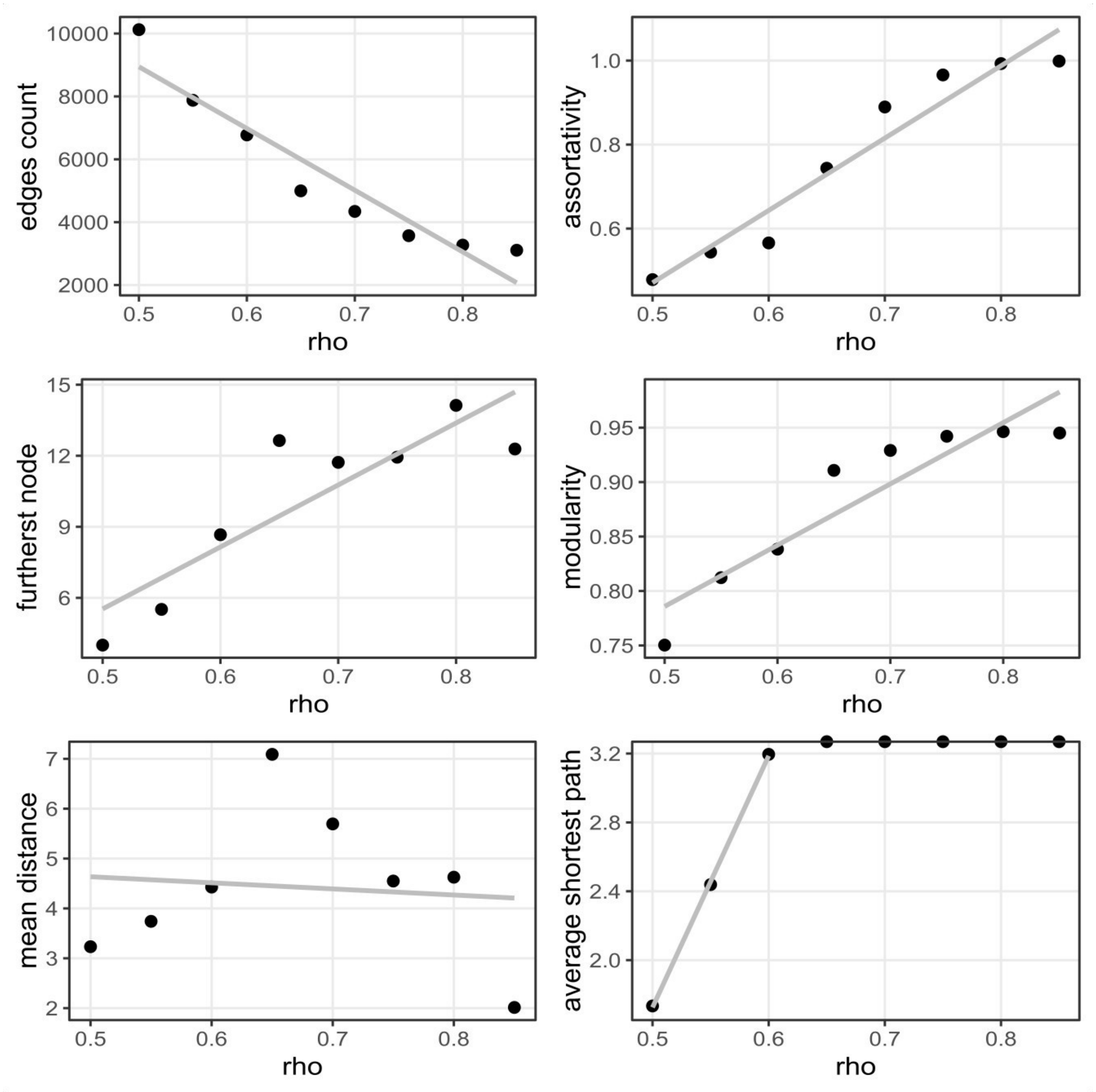

Supplementary Figure 3. Changes in network statistics as a function of increasing correlation thresholds. A linear model was fitted to the data (grey line). A clear change in network properties is present for correlation thresholds ≥ 0.60.

1.2 Supplementary Tables

Supplementary Table 1. Network statistics for increasing correlation thresholds. SPL - Shortest path length.

Correlation threshold (ρ)	Edges count	Average SPL	Assortativity	Furthest node distance	Modularity (Louvein)	Mean distance
0.5	10,124	1.73	0.48	4	0.75	3.23
0.55	7,877	2.44	0.54	5.51	0.81	3.74
0.6	6,775	3.2	0.57	8.67	0.84	4.43
0.65	4,996	Inf	0.74	12.64	0.91	7.09
0.7	4,342	Inf	0.89	11.72	0.93	5.69
0.75	3,570	Inf	0.97	11.94	0.94	4.55
0.8	3,270	Inf	0.99	14.13	0.95	4.63
0.85	3,107	Inf	1	12.29	0.95	2.01
